# Immunohistochemical Localization of AT_1a_, AT_1b_, and AT_2_ Angiotensin II Receptor Subtypes in the Rat Adrenal, Pituitary, and Brain with a Perspective Commentary

**DOI:** 10.1155/2013/175428

**Published:** 2013-03-19

**Authors:** Courtney Premer, Courtney Lamondin, Ann Mitzey, Robert C. Speth, Mark S. Brownfield

**Affiliations:** ^1^Department of Comparative Biosciences and Neuroscience Training Program, University of Wisconsin, AHABS Building, Room B29, 215 Linden Drive, Madison, WI 53706, USA; ^2^Interdisciplinary Stem Cell Institute, Miller School of Medicine, University of Miami, 1501 NW 10th Avenue, suite 832, Miami, FL 33136, USA; ^3^Department of Biochemistry, University Wisconsin, Madison, WI 53706, USA; ^4^Division of Neuroscience, Oregon National Primate Research Center, Department of Physiology and Pharmacology, Oregon Health and Science University, Beaverton, OR, 97006, USA; ^5^Department of Pharmaceutical Sciences, College of Pharmacy, Nova Southeastern University, Fort Lauderdale, FL 33328, USA

## Abstract

Angiotensin II increases blood pressure and stimulates thirst and sodium appetite in the brain. It also stimulates secretion of aldosterone from the adrenal zona glomerulosa and epinephrine from the adrenal medulla. The rat has 3 subtypes of angiotensin II receptors: AT_1a_, AT_1b_, and AT_2_. mRNAs for all three subtypes occur in the adrenal and brain. To immunohistochemically differentiate these receptor subtypes, rabbits were immunized with C-terminal fragments of these subtypes to generate receptor subtype-specific antibodies. Immunofluorescence revealed AT_1a_ and AT_2_ receptors in adrenal zona glomerulosa and medulla. AT_1b_ immunofluorescence was present in the zona glomerulosa, but not the medulla. Ultrastructural immunogold labeling for the AT_1a_ receptor in glomerulosa and medullary cells localized it to plasma membrane, endocytic vesicles, multivesicular bodies, and the nucleus. AT_1b_ and AT_2_, but not AT_1a_, immunofluorescence was observed in the anterior pituitary. Stellate cells were AT_1b_ positive while ovoid cells were AT_2_ positive. In the brain, neurons were AT_1a_, AT_1b_, and AT_2_ positive, but glia was only AT_1b_ positive. Highest levels of AT_1a_, AT_1b_, and AT_2_ receptor immunofluorescence were in the subfornical organ, median eminence, area postrema, paraventricular nucleus, and solitary tract nucleus. These studies complement those employing different techniques to characterize Ang II receptors.

## 1. Introduction

The ability of angiotensins II (Ang II) and III (Ang III) to stimulate aldosterone [1, 2] and epinephrine [3] release from the adrenal gland is well established. The central nervous system and adenohypophyseal effects of these peptides are also well documented and numerous. While the effects of Ang II on the adrenal are thought to arise primarily from blood-borne Ang II, it is clear that there is a local brain angiotensinergic system as illustrated by biochemical, immunohistochemical, behavioral, physiological, and receptor binding studies [4–8] and reviews [9–11]. The anterior pituitary also appears to be subject to both blood-borne and local angiotensinergic systems, as well as receiving indirect regulatory signals from brain angiotensinergic activity [12, 13].

In mammals, there are two primary Ang II receptor subtypes, AT_1_ and AT_2_ [14–19]. With the discovery of these multiple subtypes of Ang II receptors, pharmacological studies revealed that the AT_1_ subtype mediated both aldosterone [20] and epinephrine [21] release as well as pressor [22, 23], dipsogenic [22–24], and sodium appetite [24–26] responses to Ang II. The localization of AT_1_ receptors in the rat brain regions mediating pressor and dipsogenic actions of Ang II, such as the subfornical organ (SFO), median preoptic nucleus (MnPO), organum vasculosum of the lamina terminalis (OVLT) paraventricular nucleus of the hypothalamus (PVN), nucleus of the solitary tract (NTS), and area postrema [27–29] is consistent with this role. In contrast, AT_2_ receptors tend to be distributed in sensory, motor, and emotional regions of the brain, for example, superior colliculus, medial geniculate nucleus, locus coeruleus, lateral septum, medial amygdala, subthalamic nucleus, and inferior olivary nucleus [27–29]. It has been suggested that the medial amygdala can mediate salt appetite [30], but beyond that, the functional significance of the AT_2_ in the brain and the adrenal has not been established.

The subsequent discovery that rodents express two subtypes or isoforms of the AT_1_ receptor, AT_1a_ and AT_1b_, [31–33] raises the question as to which of these two subtypes may be mediating adrenal hormone release and the physiological effects of Ang II in the brain and pituitary. Pharmacological studies of the ability of angiotensins and AT_1_ receptor-selective antagonists to bind to the AT_1a_ and AT_1b_ receptor subtypes reveal little difference in their affinities for these two subtypes [34–37].

 PCR amplification of AT_1a_ and AT_1b_ mRNA in female rat adrenal, lung, vascular smooth muscle, pituitary, and brain indicated that the AT_1a_ subtype mRNA was predominant in the lung, vascular smooth muscle, and hypothalamus, while the AT_1b_ subtype was predominant in the adrenal, pituitary, subfornical organ, and organum vasculosum of the lamina terminalis [31, 38]. Both PCR amplification [31, 35, 38–40] and in situ hybridization [39, 41, 42] have been used to compare the expression of mRNA for these two subtypes in the adrenal and brain. However, the expression of mRNA does not always correspond with the expression of the protein it encodes. For example, estrogen treatment can reduce AT_1_ receptor expression without altering AT_1_ mRNA expression presumably via posttranscriptional inhibition of mRNA translation [43]. Moreover, in neuronal tissues, the receptors may be expressed on axonal terminals distant from their perikaryal mRNA. 

Studies of AT_1a_ and AT_1b_ mRNA expression in the adrenal indicate that the AT_1b_ subtype mRNA is predominant in the rat adrenal [35, 38, 39, 44], but that it is absent in the adrenal medulla [44–46]. Studies of AT_1a_ and AT_1b_ mRNA in rodent brain vary considerably along a continuum from a predominance of AT_1b_ expression in the female rat brain [31], to a moderate predominance of AT_1a_ in the male mouse brain [40, 42], a differential distribution of the mRNAs in a two-week-old male rat brain [45], to very low expression of AT_1b_ mRNA in the adult male rat brain [41], and to no expression of AT_1b_ mRNA in rat brain [47]. In comprehensive studies of the distribution of AT_1a_ and AT_1b_ mRNA the rat brain and pituitary [41], the AT_1a_ mRNA was found to be highly expressed in brain regions reported to mediate cardiovascular effects of Ang II, while AT_1b_ expression was very low in these regions. Conversely, AT_1b_ mRNA was very high in the anterior pituitary while AT_1a_ mRNA was low. 

To determine if the distribution of AT_1a_, AT_1b_, and AT_2_ receptor subtype protein in the rat adrenal, pituitary, and brain corresponds to the distribution of the mRNAs for these subtypes, this study uses fluorescence immunohistochemistry with antibodies directed at unique peptide fragments of each of these three subtypes to localize these receptors. 

## 2. Materials and Methods

### 2.1. Antibody Preparation

Antipeptide antibodies were generated against fragments of rat AT_1a_,AT_1b_, and AT_2_ receptors. Peptides candidates were selected by computer analysis of full length receptors retrieved from the NCBI protein database (http://www.ncbi.nlm.nih.gov/protein) and by Hopp-Woods analysis [48] for optimal antigenicity. Peptides corresponding to receptor fragments near the carboxy terminal tail of the receptor subtypes where there is a 2 amino acid difference were synthesized by solid phase peptide synthesis. For the AT_1a_ receptor, the peptide was PSDNMSSSAKKPASC, which corresponds to amino acids 341–355 of this 359 amino acid protein. For the AT_1b_ receptor, the peptide was SSSAKKSASFFEVE, which corresponds to amino acids 346–359 of this 359 amino acid protein. For the AT_2_ receptor, the peptide was CRKSSSLREMETFVS, which corresponds to amino acids 349–363 of this 363 amino acid protein (except that it contained a glutamic acid in position 358 versus an aspartic acid). The peptides were compared with the protein database (http://blast.ncbi.nlm.nih.gov/Blast.cgi) to establish the uniqueness of the peptide sequences from other known proteins.

Peptides were conjugated to keyhole limpet hemocyanin (KLH) and injected into rabbits at approximately monthly intervals for 6 months. Serum was obtained from the rabbits and affinity purified. To obtain AT_1a_-selective antibodies, serum from rabbits immunized with the peptide corresponding to the AT_1a_ receptor subtype was affinity purified using chromatography resin cross-linked with the AT_1a_ peptide. Antibodies retained by this resin were eluted with a high salt solution and the eluate was then applied to an affinity column made by cross-linking the AT_1b_ receptor peptide antigen to chromatography resin. Antibody that was not retained by the AT_1b_ resin was denoted as AT_1a_ receptor selective. Antibody that was retained by both the AT_1a_ and AT_1b_ resins was defined as nonselective for AT_1a_ or AT_1b_-receptors. A similar strategy was used to derive AT_1b_ selective antibodies except that serum from rabbits immunized with the peptide corresponding to the AT_1b_ receptor subtype was affinity purified using chromatography resin cross-linked with the AT_1b_ peptide initially. Antibodies retained by the AT_1b_ resin were subsequently applied to the AT_1a_ resin. Antibodies retained by the AT_1b_, but not the AT_1a_ resin, were classified as AT_1b_ selective. AT_2_ receptor antibodies were affinity purified using chromatography resin cross-linked with the AT_2_ receptor peptide used to generate the antibody. Antibodies retained by the AT_2_ resin were eluted with high salt solution and classified as AT_2_ selective.

### 2.2. Animals

Adult male Sprague-Dawley rats (225–300 g body weight; Harlan, Sprague Dawley) were kept in an AAALAC approved vivarium (12 : 12 Light : Dark). Standard lab chow and water were available ad lib. Animals were kept in the vivarium for at least two weeks prior to use and were housed two per cage. All procedures were approved by the University of Wisconsin, School of Veterinary Medicine Animal Care Committee.

### 2.3. Western Immunoblotting

Fresh or frozen, whole or dissected rat adrenals (*n* = 4) were employed. A 2 mm slab was cut from the center of the adrenal and the medulla was removed by punch. The cortex was dissected away from the medulla. Tissues were homogenized in one complete mini protease inhibitor tablet (Roche, Indianapolis, IN) dissolved in 7 mL of RIPA buffer (Millipore, Billerica, MD). Lysates were sonicated for 5 minutes and cleared of debris by centrifugation at 15000 rpm for 20 minutes. Samples were normalized so as to amount of protein present via BCA assay (Thermo Scientific, Rockford, IL).

 Samples were dissolved 1 : 1 in loading buffer with beta mercaptoethanol and boiled at 95°C for 4 minutes before loading. Proteins were separated via SDS-PAGE and transferred to PDVF membrane (Bio-Rad, Hercules, CA). Transfer conditions were wet (1 hour at 100 volts). Membranes were incubated for one hour in tris buffered saline containing 0.05% tween-20 (TBST), 5% powdered milk, and 1% bovine serum albumin. Blots were incubated in primary antibodies overnight at 4°C. Primary antibodies (Table 1) were diluted in TBST with 0.2% NaN_3_ as a preservative. Blots were incubated in secondary antibody for 45 minutes. Secondary antibody goat anti-rabbit HRP (KPL, Gaithersburg, MD) was diluted 1 : 100,000 in 20 mL TBST with 2 uL streptavidin HRP (Sigma Aldrich, St Louis, MO). Developing solutions used in this study were LumiGLO immunoblotting reagent (KPL) and Supersignal West Pico Substrate (Thermo Scientific).

### 2.4. Tissue Preparation

Rats were deeply anesthetized with isoflurane or pentobarbital (65 mg/kg IP) and perfused intracardially with physiological flush solution (Tyrode's solution) containing heparin and procaine followed by histological fixative (4% paraformaldehyde with 0.05% glutaraldehyde in 0.1 M sodium phosphate, pH 7.5). Brains, pituitaries, and adrenals were removed and immersion fixed at 4°C in the same solution overnight and then stored in saline until sectioning at 50 micron thickness for immunofluorescence microscopy using a Lancer vibratome. 

### 2.5. Immunofluorescence Histochemistry

 Adrenals, pituitaries, and brains from 12 rats were used for these studies. Initially all antibodies were screened at dilutions of 1 : 100 to 1 : 10,0000 in ICC buffer (PBS with 0.25% gelatin, 2% normal goat serum 0.1% thimerosal, and 0.05% neomycin) to determine working dilutions demonstrating the highest signal and lowest background signal for each tissue. Working dilutions of angiotensin II receptor antibodies were (1 : 500) primary antibody (AT_1a_, AT_1b_) and 1 : 2000 AT_2_ for 18–72 hours at 4°C. Control sections were incubated with primary antibodies incubated with an excess of the antigenic peptides (20 *μ*g/mL of antigenic peptide at the working dilution). Also antibodies were immunoprecipitated from their working dilutions by incubation with 100 *μ*L AT_1a  _, AT_1b_, or AT_2_ affinity gels and then the supernatant was used in place of the antibody solution. Sections were then incubated with Cy3-labeled goat anti-rabbit IgG and then mounted onto poly-L-lysine slides. Slides were viewed and analyzed utilizing a Nikon Eclipse E600 epifluorescence microscope with UV illumination, and a digital camera (Spot RT, Diagnostic Products).

### 2.6. Immunoelectron Microscopy

 Adrenals from 7 rats were used for ultrastructural immunocytochemistry (*N* = 4 rats for immunogold detection and *n* = 3 rats for peroxidase. For both methods, rats were perfused as described above and postfixed for 24 hours in 4% paraformaldehyde with 0.1% glutaraldehyde, washed in PBS and vibratome sectioned at 50 micron thickness. The sections were incubated in 0.1% sodium borohydride 15 minutes, permeabilized in 0.05% triton for one hour, and blocked in either 0.5% BSAc (Aurion, Arnhem, Gelderland, The Netherlands) for one hour for immunogold detection or ICC buffer for immunoperoxidase detection prior to overnight exposure to primary antibody. The primary antibody dilution for AT_1a_ receptors was 1 : 500 for both immunogold and immunoperoxidase. 

For the immunogold method antibody-labeled receptor was detected using ultrasmall gold (Aurion, 0.8 nanometer average size) diluted 1 : 100 in phosphate buffer and incubated overnight. Tissues were then postfixed in 2.5% glutaraldehyde for 30 minutes. The immunological signal was silver intensified by incubation in R-Gent SE-EM (Aurion) for one hour. For immunoperoxidase detection antibody-bound receptor was incubated with peroxidase labeled goat anti-rabbit IgG-Fab (1 : 250 overnight in the refrigerator). Peroxidase signal was visualized by incubation in diaminobenzidine (30 mg %) and hydrogen peroxide (0.01%) for 10 minutes in 0.1 M Tris HCL, pH 7.5. Then both immunogold and immunoperoxidase sections were rinsed in 0.1 M sodium phosphate buffer, fixed with osmium, dehydrated through an alcohol series to propylene oxide, and flat embedded in EMBED 812 resin (Electron Microscopy Sciences, Hatfield, PA).

Ultrathin sections were cut and adsorbed to grids coated with Formvar film (Electron Microscopy Sciences), and contrasted with uranyl acetate and lead citrate. All samples were examined and photographed with a Philips CM 120 STEM electron microscope and a Megaview 3 SIs digital camera (Olympus, Munster, Westphalia, Germany) in combination with the software program iTEM (Olympus) at the University of Wisconsin Madison Electron Microscope Facility.

## 3. Results

**Figure 1:**
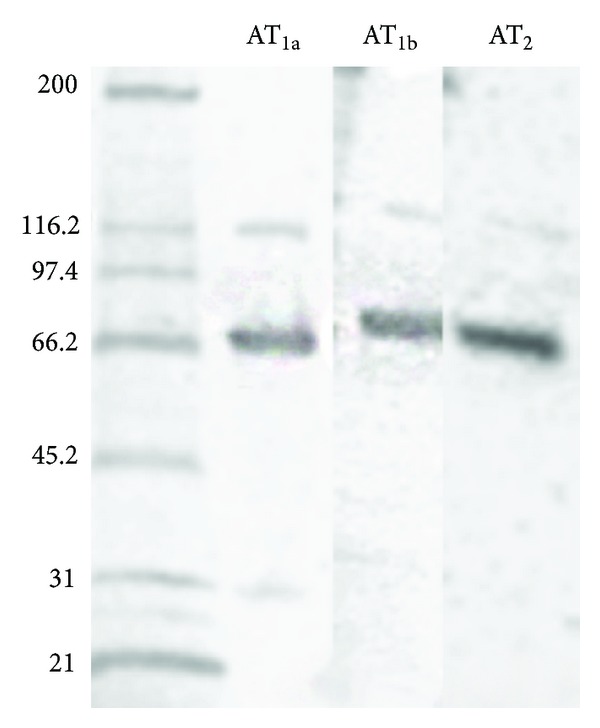
Western immunoblots for AT_1a_, AT_1b_, and AT_2_ receptors of crude extracts of whole adrenals. The three receptors show major bands at ~69–75 kD and well as faint bands at about ~116–126 kD. AT_1a_ receptor-directed antibody (Rabbit 92578-sel), AT_1b_ receptor-directed antibody (Rabbit 92587-sel), AT_2_ receptor-directed antibody (Rabbit 92595).

Western blotting of protein extracts of the adrenal with the 3 antibodies revealed primary ~69, ~75, and ~71 kD bands for the AT_1a  _, AT_1b_, and AT_2_ receptors, respectively, with secondary bands of ~116, ~126, and ~119, respectively (Figure 1). This suggests that the solubilized receptor was glycosylated since the theoretical molecular weights of the deglycosylated receptors are 40759 Daltons for the AT_1a_, 40781 Daltons for the AT_1b_, and 41200 Daltons for the AT_2_ receptor. The secondary bands most likely represent dimerized receptors or receptor-protein complexes.

**Figure 2:**
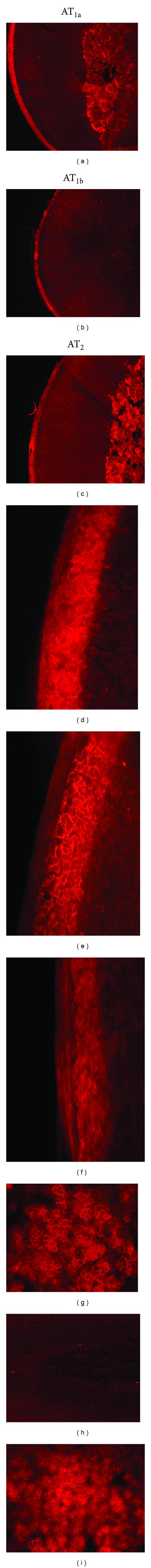
Immunofluorescent localization of AT_1a_, AT_1b_, and AT_2_ receptors in rat adrenals. Survey photomicrographs show positive immunofluorescence for AT_1a_ ((a) and (d); 80x), AT_1b_ ((b) and (e)), and AT_2_ ((c) and (f)) in the zona glomerulosa (160x). Positive staining for AT_1a_ ((a) and (g)) and AT_2_ ((c) and (i)), but not AT_1b_ ((b) and (h)), is present in the adrenal medulla. The antibodies used were those used in Figure 1.

Immunofluorescent staining of the adrenal with the 3 antibodies gave differing discrete staining patterns in the adrenal. Using a working dilution of 1 : 500 AT_1a_, immunoreactivity was seen in both the adrenal medulla and the zona glomerulosa (Figures 2(a), 2(d), and 2(g)). The staining was primarily cytoplasmic in both regions, although in the medulla, localization to the cell membrane is apparent in some cells (Figure 2(g)). AT_1b_ immunoreactivity was present in abundance in the zona glomerulosa of the adrenal (Figures 2(b) and 2(e)). The immunofluorescence was primarily localized to the cell membrane (Figure 2(e)). Weak AT_1b_ immunoreactivity was also present in the zona reticulata (Figure 2(e)). AT_1b_ immunostaining was nearly nonexistent in the medulla (Figure 2(h)). 

AT_2_ immunoreactivity was abundantly present in both the adrenal medulla and the zona glomerulosa (Figures 2(c), 2(f), and 2(i)). The AT_2_ immunofluorescence was also primarily cytoplasmic although a plasma membrane localization was seen in many medullary cells (Figure 2(i)). No immunofluorescent signal was seen in any sections incubated with the antigenic peptide preadsorbed antibodies (not shown).

**Figure 3:**
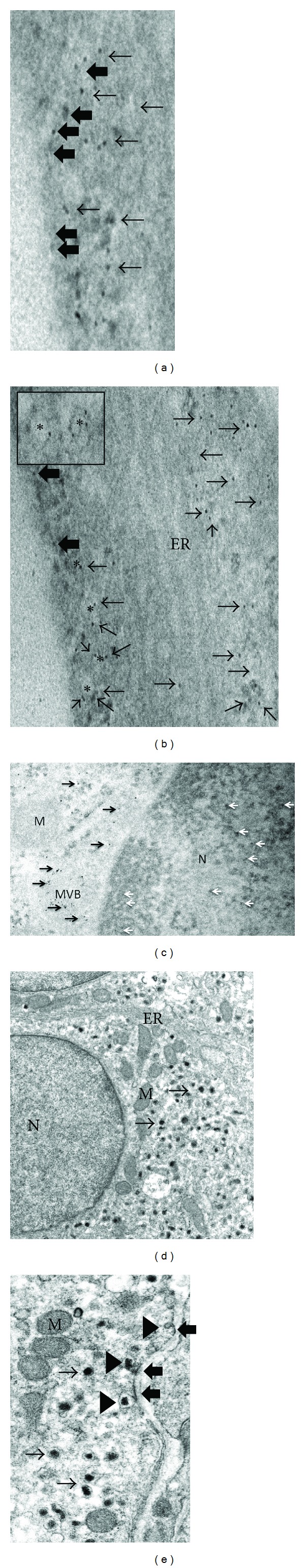
Ultrastructural immunocytochemistry of the AT_1a_ receptor (using anti-AT_1a_ receptor #92578-sel in zona glomerulosa (Figures 3(a) through 3(c); 48,000x) and adrenal medulla (Figures 3(d) and 3(e)). Immunogold ultrastructural analysis of zona glomerulosa shows AT_1a_ receptor (showing localization at the cell membrane (bold arrows; Figures 3(a) and 3(b)), in the cytoplasm (line arrows; Figures 3(a) through 3(c)), and on the surface of endocytic vesicles (insert Figure 3(b)). Immunogold particles were also seen in a multivesicular body (MVB) and in the nucleus (Figure 3(c)). Immunoperoxidase staining of adrenal medullary cells reveals a large number of AT_1a_ positive vesicles (line arrows; Figure 3(d); 20,000x and Figure 3(e); 48,000x), patches of membrane receptors (block arrows; Figure 3(e)), and apparent omega body fusion with the cell membrane (arrow heads; Figure 3(e)). Note the lack of localization in the mitochondria (M) and endoplasmic reticulum (ER).

Immunoelectron microscopic analysis of the subcellular localization of AT_1a_ receptors in the zona glomerulosa and medulla is shown in Figure 3. Both cell membrane and cytoplasmic labeling for AT_1a_ receptors was seen in these cells. AT_1a_ receptor immunogold labeling of endocytic vesicles and mature multivesicular vesicular bodies was seen in glomerulosa cells (Figures 3(b) and 3(c)) and immunoperoxidase labeling of cell membrane and newly forming endocytic vesicles was seen in medullary cells (Figure 3(e)). Intranuclear AT_1a_ receptor immunogold staining was observed in cells of the zona glomerulosa. However, AT_1a_ receptor-immunogold staining was not evident in mitochondria or endoplasmic reticulum of either glomerulosa or medullary cells. 

**Figure 4:**
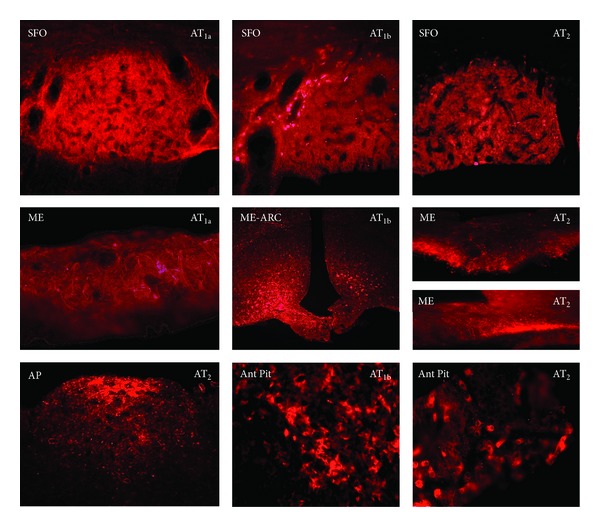
Circumventricular organs and pituitary AT receptor immunolocalization. Subfornical organ (top row left to right; 180x) AT_1a_, AT_1b_, and AT_2_. Median eminence (middle row left to right AT_1a_ (160x), AT_1b_ (100x), and AT_2_ (120x)). Bottom row area postrema (AP, 160x) AT_2_ and anterior pituitary (pars distalis localization; 120x) of AT_1b_ (middle) and AT_2_ (right). No staining for AT_1a_ was seen in the anterior pituitary.

AT_1b_ immunoreactivity was observed in the pars distalis of the anterior pituitary. It was primarily localized to stellate cells, but significant numbers of ovoid cells were also immunopositive. By contrast, AT_1a_ immunoreactivity was not observed in the pituitary (Figure 4). AT_2_ receptor immunoreactivity also was observed in the pars distalis of the anterior pituitary, primarily in ovoid cells. AT_1a_ and AT_1b_ receptor immunoreactivity was observed on nerve fibers in the posterior pituitary (Table 1). No Ang II receptor immunoreactivity was observed in the intermediate lobe of the pituitary.

**Figure 5:**
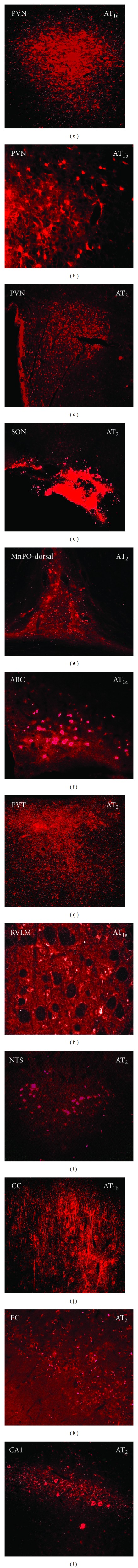
Immunofluorescent localization of AT receptors in various brain nuclei. Top row: hypothalamic paraventricular nucleus (PVN, 160x) AT_1a_, AT_1b_, and AT_2_. Second row: immunofluorescence labeling (left to right) for AT_2_ in supraoptic nucleus (SON; 120x) and median preoptic nucleus (MnPO-dorsal part: 80x), and AT_1a_ localization labeling of arcuate nucleus (ARC; 160x). Third row, (left to right): AT_2_ in the periventricular nucleus of the thalamus (PVT; 160x), AT_1a_ receptor in the rostral ventrolateral medulla (RVLM, 100x), and AT_2_ receptor in nucleus of the solitary tract (NTS; 160x). Bottom row (left to right): AT_1a_ in frontal parietal cortex (160x), AT_2_ in entorhinal cortex (80x), and AT_2_ in hippocampus CA1 (120x).

**Figure 6:**
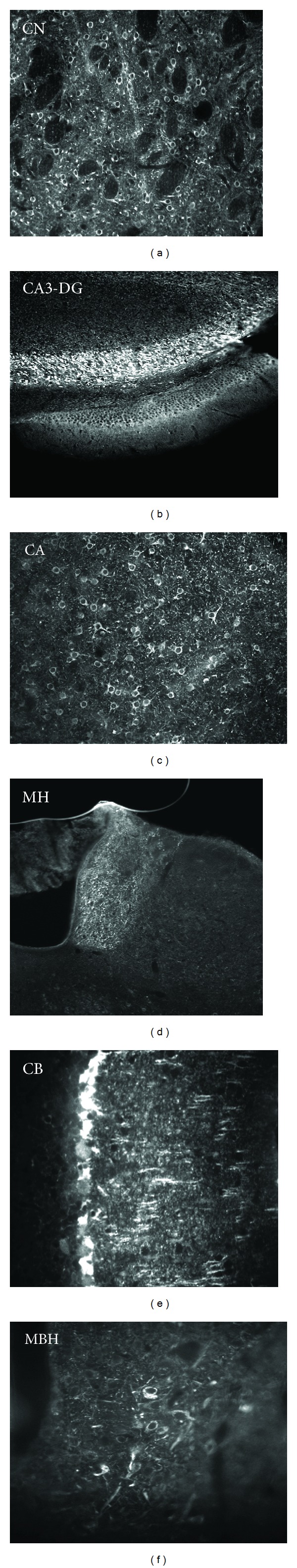
AT_2_ receptor immunolocalization in caudate nucleus (CN, 400x), CA3-dentate gyrus of the hippocampus (CA3-DG; 80x), central nucleus of the amygdala (CA; 160x), medial habenula (MH, bottom left; 80x), AT_1b_ in cerebellar Purkinje cells (CB; bottom middle; 100x), and AT_2_ in mediobasal hypothalamus (MBH; bottom right, 320x).

**Figure 7:**
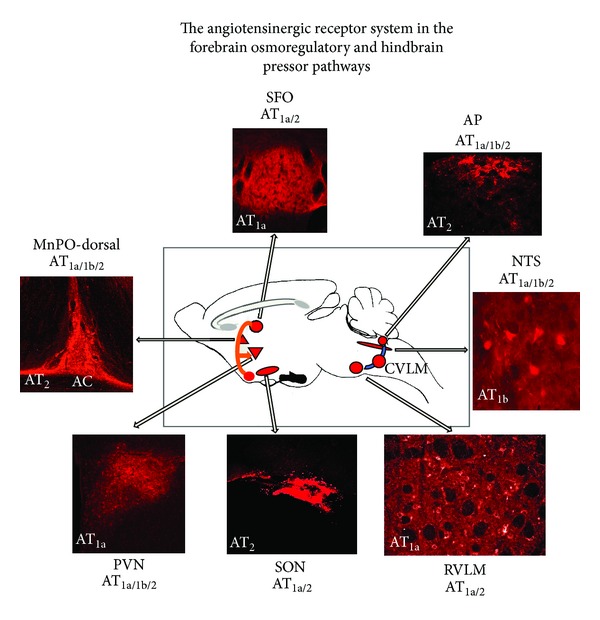
Diagrammatic summary of important brain nuclei in the angiotensinergic forebrain osmoregulatory pressor (orange pathway) and hindbrain pressor (blue) pathways. Note that there is more than one AT receptor in each site as given adjacent to each micrograph. A representative AT receptor for each site is shown within the figure.

 In sections from the brain, neurons were immunopositive for all three receptors, but glial cells showing astrocytic (and microglial, Figure 4 center panel) characteristics were immunopositive only for AT_1b_. Immunoreactivity for all three angiotensin receptor subtypes was present in abundance in brain regions reported to have high angiotensin receptor density by ligand binding studies and other immunohistochemistry studies (Figures 4–7, Table 1). These regions include the SFO, median eminence, PVN, NTS, and area postrema (Figures 4 and 5, Table 1). In all five of these locations, we demonstrated the presence of all three receptors, although their distribution within each region was not identical (Table 1). Of note, AT_1b_ receptor immunoreactivity was present in the magnocellular division of the PVN while AT_2_ receptor immunoreactivity was present in the supraoptic nucleus (SON) (Figure 5). AT_2_ receptors were more widely distributed than AT_1a_ and AT_1b_ receptors in the brain, and their immunoreactivity was found in every region in which AT_1_ receptor immunoreactivity was observed (Table 1). AT_2_ receptor immunoreactivity was found exclusively in the amygdala, piriform cortex, thalamus, and medial epithalamus (Figures 5 and 6, Table 1).

Angiotensin II receptor immunoreactivity also was found in rat brain regions generally reported to have low expression of Ang II receptors. These include neurons in the cerebral cortex (AT_1b_ and AT_2_), hippocampus (AT_1a_ and AT_2_), caudate nucleus (AT_1a  _, AT_1b_ and AT_2_), and SON (AT_2_) (Figure 5). 

## 4. Discussion

### 4.1. Antibody Development Strategy

The results of these studies unequivocally demonstrate a differential distribution of AT_1a  _, AT_1b_, and AT_2_ receptor immunostaining. This was accomplished by precise epitope targeting within the C-terminus of each receptor, selective antipeptide affinity chromatographic purification methods, Western blotting, and tissue specificity studies in adrenal and pituitary where the distribution of these AT receptor-expressing cells has been established by in situ hybridization and receptor binding studies. 

The initial identification of the two subtypes of AT_1_ Ang II receptors in rodents demonstrated the presence of mRNA for both the AT_1a_ and AT_1b_ subtype in the rat adrenals [32, 38, 49]. The AT_1b_ was identified as the predominant AT_1_ receptor subtype in the rat adrenal based on mRNA expression [38, 49]. While these initial observations have been confirmed in the rat adrenal [50, 51], the AT_1a_ is considered to be the predominant AT_1_ receptor subtype in all other rat tissues except the anterior pituitary based on mRNA expression [38, 52].

It is important to be able to discriminate AT_1a_ and AT_1b_ receptor protein expression, because their mRNAs are differentially regulated [31, 39, 49, 52–54]. Furthermore, it is important to determine if the changes in mRNA expression translate into changes in expression of these receptor subtypes, because mRNA expression does not always correlate with protein expression. For example, in the kidney losartan increases AT_1a_ receptor mRNA expression, but decreases AT_1_ receptor binding [55]. The existence of miRNAs for angiotensin receptors, for example, miR-155 [56] further erodes the value of mRNA levels as indicators of angiotensin receptor protein expression. Functionality of the subtypes may also differ; AT_1a_ and AT_1b_ can stimulate aldosterone release, while AT_1a_, but not AT_1b_, can stimulate corticosterone release in the mouse adrenal [57]. 

 In view of the near identical pharmacological characteristics of the AT_1a_ and AT_1b_ receptor subtypes [34–36], the only way to discriminate these two proteins is to exploit immunological differences arising from differences in their amino acid sequences. While the AT_1a_ receptor (accession no. P25095, http://www.ncbi.nlm.nih.gov/protein/113493 (accessed 16 March 2012) and AT_1b_ receptor (accession no. NP 112271) http://www.ncbi.nlm.nih.gov/protein/82524858NP112271 (accessed 16 March 2012) subtypes are encoded by separate genes, they are ~95% identical and are both made up of 359 amino acids [33, 38]. Thus there are only a few regions of these receptors where they differ substantially in amino acid sequence. One of these regions, near the carboxy terminus of the receptor proteins (amino acids 352 to 355), has 2 different amino acids in this 4 amino acid stretch. The closest similarities to the sequences of the AT_1_ antigenic peptides in the protein database (Protein Blast) http://blast.ncbi.nlm.nih.gov/Blast.cgi?PROGRAM=blastp&BLASTPROGRAMS=blastp&PAGETYPE=BlastSearch&SHOWDEFAULTS=on&LINKLOC=blasthome, accessed on February 4, 2013) were the serotonin 5 HT2b subtype with a 7 amino acid identity to the AT_1a_ peptide fragment (score = 24.0 bits) and sestrin 1 with a 7 amino acid identity to the AT_1b_ peptide fragment (score = 24.4 bits). 

To generate an antibody to the AT_2_ receptor, a similar strategy was applied. A C-terminal domain peptide of 15 amino acids (resembling amino acids 349 to 363) was used as the antigen. The sequence of the AT_2_ receptor (accession no. P35351, http://www.ncbi.nlm.nih.gov/protein/543780 accessed on February 4, 2013) has negligible homology with either of the AT_1_ receptor subtypes. The closest similarity to this peptide sequence was an immunoglobulin kappa chain (AAA41415.1) with an 8 amino acid identity to the AT_2_ peptide fragment (score = 27.4 bits compared to 49.0 bits for the AT_2_ receptor).

### 4.2. Adrenal AT Receptor Subtype Localization

 The presence of AT_1a_,   AT_1b_, and AT_2_ angiotensin receptor subtype immunoreactivity in the rat adrenal was clearly demonstrated in this study. AT_1a_ and AT_2_ receptor subtype immunoreactivities were found in both the zona glomerulosa and medulla, which is consistent with receptor binding studies [37, 58–61] and mRNA studies [44, 61–64]. AT_1b_ receptor was not observed in the adrenal medulla, but was present in the zona glomerulosa. This is consistent with in situ hybridization studies of the distribution of AT_1b_ mRNA in the adrenal [39, 44, 46, 54]. 

Other studies of the localization of Ang II receptor subtype immunoreactivity in the adrenal have given mixed and controversial results. Paxton et al. [65] observed AT_1_ receptor immunoreactivity in the zona glomerulosa of the rat adrenal with an antibody prepared against amino acids 15–24 of the rat AT_1a_ and AT_1b_ receptor. However, they did not observe any AT_1_ receptor immunoreactivity in the adrenal medulla. Similarly, Lehoux et al. [66] observed AT_1_ immunoreactivity in the zona glomerulosa of the rat adrenal cortex, but not in the medulla using an antibody raised against amino acids 306–359 of the human AT_1_ receptor subtype. Of note, adrenals from rats kept on a low sodium diet displayed AT_1_ immunoreactivity in other cortical zones (fasciculata and reticularis). The lack of adrenomedullary staining with this human antibody suggests that it may only recognize the AT_1b_ sequence in the rat. Giles et al. [67] observed AT_1_ immunoreactivity and AT_1_ mRNA in the zona glomerulosa of rat adrenals using an antibody directed against amino acids 350–359 of the rat AT_1a_ subtype plus a small amount of immunoreactivity in the zona fasciculata. However, there was no mention of AT_1_ immunoreactivity or mRNA in the adrenal medulla. 

Frei et al. [68] observed AT_1_ immunoreactivity in the rat adrenal cortex and medulla using a monoclonal antibody raised against amino acids 229–246 of the human AT_1_ receptor subtype. Yet, AT_2_ immunoreactivity was only observed in the rat adrenal medulla using an antibody raised against amino acids 314–330 of the human AT_2_ receptor subtype. On the other hand, Harada et al. [63] observed AT_2_ receptor immunoreactivity in immunoblots of the rat adrenal cortex, but not in the medulla using two different antibodies—one raised against amino acids 21–35 of the rat AT_2_ receptor and one raised against amino acids 221–363 of the human AT_2_ receptor. However, they did detect a low level of AT_2_ receptor-like immunoreactivity in the medulla using the latter antibody for immunohistochemical analysis. Conversely, Yiu et al. [69] reported AT_2_ immunoreactivity only in the rat adrenal medulla using an antibody directed against amino acids 341–351 of the rat AT_2_ subtype. Notably, they reported that this antibody failed to label brain regions known to express AT_2_ receptors. Reagan et al. [70] were unable to demonstrate any AT_2_ receptor immunoreactivity in the rat adrenal using a polyclonal antibody developed to recognize AT_2_ receptors in N1E-115 cells. 

### 4.3. Subcellular Localization of AT_1_ Receptors

Localization of immunofluorescence for all three angiotensin receptor subtypes to the cell membrane as well as the cytoplasm in the adrenal is consistent with the behavior of other G protein coupled receptors that are functionally expressed on cell membranes but undergo receptor-mediated internalization [71]. The electron microscopic localization of AT_1a_ immunoreactivity to putative developing endosomes still in contact with the cell membrane (Figure 3(e)) is consistent with receptor mediated endocytosis as the mechanism of angiotensin receptor internalization [72]. In addition, there is now a considerable body of evidence supporting the existence of an intracellular RAS which signals via AT_1_ receptors [73].

Noteworthy in our study is the nuclear localization of adrenal AT_1a_ receptors. The ability of G protein coupled receptors to localize and signal directly to the cell nucleus is firmly established [74] and likely includes angiotensin receptors. Beginning with the electron microscopic studies localizing ^3^H-Ang II to myocardial cell nuclei [75], it has been suspected that Ang II receptors are present in cell nuclei. The existence of nuclear Ang II receptors was subsequently documented in isolated hepatic nuclei by Re and Parab [76] who showed that Ang II increased RNA polymerase II activity, increasing RNA synthesis. Notably, they used 4 mM dithiothreitol an inhibitor of Ang II binding to AT_1_ receptors [77], suggesting that the Ang II effect might be mediated by AT_2_ receptors. Eggena et al. [78] showed that AT_1_ receptor subtype binding was present in rat hepatic cell nuclei and that Ang II could specifically induce transcription of mRNA for renin and angiotensinogen in isolated rat liver nuclei. Moreover, hepatic nuclear AT_1_ receptor binding and functionality could be dynamically regulated by adrenalectomy and nephrectomy [79]. Re et al. [80] and Eggena et al. [79] reported that nuclear Ang II receptor binding was associated with nuclear chromatin. Of note, Re et al. [80] observed ^125^I-Ang II binding to nuclear chromatin in the presence of 5 mM dithiothreitol, again suggesting that ^125^I-Ang II may be binding to AT_2_ receptors [15, 77]. The relative abundance of AT_1a_ binding within the nucleus, but not the nuclear membrane of the glomerulosa cells in this study, is consistent with localization to nuclear chromatin. AT_1_ receptor binding sites have also been identified in rat hepatocyte nuclear membranes by Booz et al. [81] and Tang et al. [82]. Interestingly, Tang et al. [82] determined that the majority of the AT1-like binding of Ang II in hepatocyte nuclei was bound to a soluble intranuclear protein. Licea et al. [83] demonstrated nuclear Ang II receptor binding in nuclei of rat renal cortex. Tadevosyan et al. [84] showed that Ang II could stimulate *α*-^32^P-UTP incorporation into RNA and increase NF-kappaB mRNA expression in isolated rat heart cardiomyocyte nuclei suggesting a nuclear site of action of Ang II.

Additional evidence supporting a nuclear localization of angiotensin receptors includes studies using an AT_1_ receptor-GFP fusion construct which translocates to the nucleus in Chinese hamster ovary cells [85] and human embryonic kidney (HEK-293) cells [86], as well as immunohistochemical studies showing colocalization of AT_1_ and AT_2_ immunoreactivity with the nuclear membrane markers nucleoporin-62 and histone-3 [84]. Moreover, the AT_1_ receptor contains a nuclear localization signal motif (KKFKK, 307-11) in its intracellular carboxy terminal tail [87], which promotes its translocation to the cell nucleus. Mutation of one amino acid in this motif (K307Q) in an AT_1a_ r-GFP receptor construct prevents it from localizing to the nucleus of HEK293 cells [86]. Of note, both agonist induced [87] and agonist independent [71, 88] nuclear localization of AT_1_ receptors has been reported.

While there are no published reports of adrenal nuclear angiotensin receptor binding or function, Eggena et al. [78] reported preliminary data suggesting that Ang II could stimulate RNA transcription in isolated adrenal nuclei. In addition, Goodfriend and Peach [89] suggested that Ang III can act intracellularly in the zona glomerulosa to promote aldosterone production. 

### 4.4. Pituitary AT Receptor Subtype Localization

Both AT_1b_ and AT_2_ receptor immunoreactivities were present in high amounts in the anterior pituitary. As noted previously mRNA for AT_1b_ receptors is abundant in the anterior pituitary, while AT_1a_ mRNA is much less abundant and AT_2_ mRNA is not observed in the anterior pituitary [47, 90]. Autoradiography and radioligand binding studies have demonstrated a high density of Ang II receptors in the anterior pituitary [37, 91–93]. This binding displays AT_1_ receptor characteristics, and little or no AT_2_ receptor binding has been observed [27, 94]. AT_1b_ expression was highest in stellate cells, while AT_2_ expression was highest in ovoid cells. Both AT_1a_ and AT_1b_ immunoreactivity was present on nerve fibers in the posterior pituitary. The ability of Ang II to affect the release of pituitary hormones is well known [95]. There are no reports of Ang II receptor binding in the posterior pituitary of the rat, although there is one report of AT_1_ receptor-immunoreactivity in nerve fibers and cell bodies in the posterior pituitary [96] and one report of AT_2_ receptor-immunoreactivity in the posterior pituitary as well as in the vasopressinergic magnocellular division of the PVN and the SON [97]. mRNA studies indicate a predominance of the AT_1b_ subtype in the anterior pituitary of the rat [38, 98–100], with little or no AT_1a_ and AT_2_ mRNA. 

Many of the pituitary hormone-releasing effects of Ang II occur in the hypothalamus and those effects are discussed below. However, some of the pituitary hormone releasing of Ang II occur directly in the pituitary. Systemically administered Ang II stimulates vasopressin release from the posterior pituitary of the dog [101, 102]; however, this may not generalize to the rat. AT_1a_ and AT_1b_ receptors on nerve fibers in the rat posterior pituitary [96] could mediate these effects of Ang II, reminiscent of the mechanism whereby Ang II acts on sympathetic nerve terminals to stimulate norepinephrine release [103, 104]. 

 Radioligand binding studies have revealed high levels of Ang II receptor binding in a lactotroph enriched pituitary preparation [105]. mRNA studies indicate that AT_1b_ receptors appear most often on lactotrophs, being present on more than 50% of all lactotrophs [98]. The appearance of AT_1b_ immunoreactivity in ovoid cells is consistent with these receptors being present on lactotrophs. It has been reported that AT_1b_ mRNA is present in a somatotroph cell line [100]. Somatotrophs are also ovoid in shape and blood-borne Ang II can inhibit growth hormone release [106], although it has also been reported that Ang II synthesized by and released from lactotrophs can stimulate the release of growth hormone from somatotrophs, [107] suggesting that somatotrophs may have excitatory AT_1_ receptors and inhibitory AT_2_ receptors. 

 ACTH release from dissociated corticotrophs in the anterior pituitary is also stimulated by Ang II *in vitro* [108]. The stimulation decreases with supraphysiological estradiol exposure *in vivo* and correlates positively with reductions in Ang II receptor binding caused by *in vivo* supraphysiological estradiol exposure [108]. Autoradiographic studies of AT_1_ receptor binding in the anterior pituitary indicate that AT_1_ receptor binding varies with the estrous cycle and that exogenous estrogen decreases anterior pituitary AT_1 _receptor binding in ovariectomized rats [109]. mRNA for AT_1b_ receptors in the anterior pituitary is also suppressed by estrogen treatment [38, 110]. The appearance of high levels of AT_1b_ immunoreactivity in stellate cells in this study is consistent with these receptors being present on corticotrophs. 

 There is one report of AT_2_ receptor immunoreactivity in pituitary adenoma blood vessels in humans [96], leading to the hypothesis that AT_2_ receptors in could participate in tumor-induced angiogenesis. 

### 4.5. Brain AT Receptor Subtype Localization

These studies describe a widespread distribution of AT_1a_, AT_1b_, and AT_2_ receptor immunoreactivity throughout the rat brain. The receptors were expressed abundantly in a number of brain regions that constitute the cardiovascular regulatory circuits of the brain, as well as the noncardiovascular regulatory regions of the brain. There was considerable variation in the degree of expression of the receptors in different regions reminiscent of the profound differences in radioligand binding for Ang II receptors, particularly among the AT_1_ receptors. AT_2_ receptors displayed an unanticipated widespread distribution throughout the rat brain, which contrasts with their limited distribution as indicated by radioligand binding studies. While AT_1_ receptors are considered to play the predominant role of mediating the actions of Ang II in the brain, AT_2_ receptors are increasingly recognized as having an important role as physiological antagonists of AT_1_ receptor effects. The codistribution of AT_1_ and AT_2_ receptors in several brain regions as well as the adrenal is consistent with the concept of colocalization of these two subtypes in the same cells as counter regulators to each other at the cellular level as well as on an organismic level [111–113]. 

The selective expression of AT_1b_ receptors on astrocytes suggests that there is a cell-specific expression of Ang II receptor subtypes in the brain. Functional AT_1_ receptors are present in primary cultures of astroglia from rat brain [114], but questions have been raised as to whether this expression could reflect an altered phenotype of cultured cells not seen in situin a living brain [115]. In contrast, Füchtbauer et al. [116] observed AT_1_ immunoreactivity (Santa Cruz, sc-579, amino acids 306–359) in astrocytes of the outer molecular layer of the dentate gyrus of the mouse brain, but did not see AT_1_ immunoreactivity in the microglia. Of note, retinal astrocytes also express AT_1_ receptor immunoreactivity (Alomone, #AAR-011 amino acids 4–18) while amacrine cells in the rat retina display AT_2_ immunoreactivity (Alomone, no. AAR12, amino acids 21–35) [117]. These reports and our observations suggest that glia do express AT_1_ receptors and that they are of the AT_1b_ subtype. Since astrocytes are the primary source of angiotensinogen in the brain, the AT_1b_ receptor may play a role in regulating angiotensinogen in the brain. 

The expression of AT_1b_ receptor immunoreactivity on cells with the morphological characteristics of microglia suggests that this receptor subtype mediates the proinflammatory effects of Ang II. AT_1_ receptor antagonism blocks the activation of microglia in an animal model of brain inflammation [118]. Proinflammatory cytokine participation in the pressor actions of Ang II in the brain is reversible by AT_1_ antagonists [119, 120], suggesting that microglial AT_1_ receptors may play a role in blood pressure regulation as well as inflammation.

The concept of the presence of Ang II receptors in the brain was firmly established by the cross-perfusion studies of Bickerton and Buckley [121] showing that blood-borne Ang II had sympathoexcitatory effects mediated by the brain. Since that time, a multitude of methodological approaches have been used to map the distribution of Ang II receptors in the brain. Early radioligand binding studies of brain Ang II receptors [122, 123] indicated that Ang II receptors were located in regions within the blood-brain barrier, for example, cerebellum, hypothalamus, thalamus, septum, and midbrain, as well as outside the blood brain barrier. The first receptor autoradiographic study of brain Ang II receptors for blood-borne Ang II clearly demonstrated their presence in 4 circumventricular organs (CVOs): the SFO, OVLT, median eminence, and area postrema [124]. *In vitro* receptor autoradiographic studies of the rat brain confirmed the localization of Ang II receptors in these CVOs and revealed a widespread distribution of discrete populations of Ang II receptors in a large number of brain nuclei [93, 125]. Subsequent receptor autoradiographic studies using Ang II receptor subtype specific competing ligands indicated that both AT_1_ and AT_2_ receptors were present in the brain and were differentially distributed [27, 58]. Regions containing high densities of AT_1_ receptor binding include regions associated with dipsogenesis and cardiovascular regulation, for example, SFO, OVLT, MnPO, PVN, NTS, dorsal motor nucleus of the vagus, area postrema, rostral ventrolateral medulla (RVLM), as well as noncardiovascular regulatory regions, for example, pyriform cortex, subiculum, and spinal trigeminal nucleus. Generally, regions containing high densities of AT_2_ receptor binding are unrelated to blood pressure regulation and dipsogenesis, for example, mediodorsal thalamus, inferior olivary nucleus, medial geniculate, and subthalamic nucleus. While many regions have a strong predominance of one or the other subtype, several brain regions show both AT_1_ and AT_2_ receptor binding, for example, parabrachial nuclei, pedunculopontine tegmental nucleus, locus coeruleus, and superior colliculus [126].

Localized injection of exogenous Ang II has been used to map the distribution of brain Ang II receptors. Early studies directed at determining sites of action of Ang II assessed its behavioral and physiological effects. Subsequent studies using iontophoretic or pressure injection of Ang II via micropipettes have focused on its cellular effects. Early mapping of Ang II receptors mediating its dipsogenic effects indicated a widespread distribution in the forebrain [127]. However, a subsequent study [128] revealed that all the active sites were targeted with a cannula that traversed the anterior cerebral ventricles, and that only when Ang II leaked into the ventricles that a dipsogenic response occurred. Microinjection of Ang II into the SFO and PVN is excitatory to these neurons [129]. Microinjection of Ang II into the RVLM [130], area postrema, and NTS [131] increases blood pressure. Microinjection of Ang II into the periaqueductal gray increases blood pressure via its actions at AT_1_ receptors [132], while microinjection of Ang II into the superior colliculus increases blood pressure via its actions at AT_2_ receptors [133] consistent with radioligand binding studies indicating the presence of AT_1_ or AT_2_ receptors in these regions [27]. Lastly, the distribution of angiotensin responsive neurons has been determined using induction of fos expression as a functional marker [134]. 

 A major controversy involves the presence or absence of Ang II receptors on vasopressinergic and oxytocinergic neurons in the SON and the magnocellular division of the PVN. Stimulation of vasopressin and oxytocin release from the posterior pituitary results from stimulation of the magnocellular neurons in the PVN and SON. In this study, all 3 Ang II receptor subtypes were highly expressed in the magnocellular divisions of the PVN. Radioligand binding studies of Ang II receptors reveal high expression of AT_1_ receptors in the parvocellular region of the PVN and low expression of Ang II receptors in the magnocellular division of the PVN and SON (as described in the previous section). Similarly, mRNA studies (succeeding section) have failed to demonstrate measurable Ang II receptor synthesizing capacity in these regions. However, electrophysiological studies suggest that neurons in these regions are responsive to Ang II. Nagatomo et al. [135] showed that Ang II inhibited potassium currents in SON neurons using patch clamping in brain slices. Ang II has a direct excitatory effect in the SON, which is consistent with the presence of AT_1_ receptors on vasopressinergic and oxytocinergic neurons [136]. The data reported herein is consistent with the presence of functional AT_1_ receptors in the PVN and SON.

 Parvocellular PVN AT_1_ receptors revealed by radioligand binding and mRNA assays are well placed to stimulate CRH neurons in the PVN to release corticotrophin releasing hormone (CRH) from their nerve terminals in the median eminence into the hypothalamo-hypophyseal portal vessels to act upon corticotrophs in the anterior pituitary. In this study, all 3 Ang II receptor subtypes were highly expressed in the parvocellular division of the PVN. 

The use of in situ hybridization or PCR for localization of mRNA to determine sites of synthesis of proteins has been widely used to localize Ang II receptor subtypes in the brain. Kakar et al. [31] reported a predominance of AT_1b_ mRNA in the SFO, OVLT, and cerebellum and a predominance of AT_1b_ in the hypothalamus by PCR. Conversely, Johren et al. [45] identified AT_1a_ mRNA in the SFO, OVLT, PVN, cerebral cortex and hippocampus, AT_1b_ mRNA in the cerebral cortex and hippocampus, (but not in the SFO or OVLT) and AT_2_ mRNA in the medial geniculate and inferior olivary nucleus. Similarly, Lenkei et al. [41] reported a predominance of AT_1a_ mRNA expression in the SFO, OVLT, PVN, and MnPO as well as the anterior olfactory nucleus with very low AT_1b_ mRNA expression in the SFO and PVN. Lenkei et al. [137] also reported the absence of AT_1a_ and AT_1b_ mRNA in the vasopressin positive neurons and GFAP positive astroglia in the SON and PVN. In the two-week-old rat brains, Jöhren and Saavedra [138] also observed AT_1a_ mRNA in the pyriform cortex, basal amygdala and choroid plexus and AT_1b_ mRNA in the choroid plexus. AT_1_ receptor binding has been reported in the choroid plexus [139] although at very low levels [140].

Brain AT_2_ receptor mRNA shows both similarities and differences from AT_2_ receptor binding in the rat brain. Noteworthy is the presence of AT_2_ mRNA in the red nucleus and the absence of AT_2_ mRNA in the locus coeruleus, lateral septum, and cerebellum [141]. These discrepancies have been interpreted as indicating that the red nucleus synthesizes AT_2_ receptors that are only expressed on its efferent nerve terminals that project to the inferior olivary nucleus and cerebellum, while the AT_2_ receptor expressing brain regions devoid of AT_2_ mRNA express AT_2_ receptors on the nerve terminals of its afferents from other brain regions. Lenkei et al. [142] observed AT_2_ mRNA in the red nucleus. However, they also observed AT_2_ mRNA in the lateral septum and locus coeruleus, as well as a much greater number of brain regions, including some traditionally AT_1_ predominant regions such as the NTS and spinal trigeminal nucleus. Lenkei et al. [47] also did a comprehensive in situ hybridization analysis of the rat brain AT_1a_ receptor mRNA. Overall this is consistent with AT_1_ receptor binding, with a few exceptions, for example, the lack of AT_1_ mRNA in arcuate nucleus and median eminence, where it is postulated that the AT_1_ receptors occur on nerve terminals of hypothalamic neurons that synthesize dopamine or releasing hormones and release them into the hypothalamo-hypophyseal portal system to act upon endocrine cells of the anterior pituitary. There are also some brain regions that express AT_1a_ mRNA, but not AT_1_ receptor binding, such as hippocampus CA1 and CA2 and some thalamic and brainstem nuclei [47]. An area of considerable cardiovascular regulatory significance is the RVLM. Chronic Ang II infusion was shown to up-regulate AT_1_ mRNA in the RVLM and reduce it in the SFO, suggesting that enhanced activation of the RVLM by enhanced AT_1 _stimulation increases sympathetic nervous system activity [143]. 

There are a large number of studies that have used immunohistochemistry and Western blotting to identify and localize Ang II receptor subtypes in the central nervous system. The receptor antigens are generally peptide fragments from different domains of the receptor protein, although one antibody [144] was generated from a purified AT_2_ receptor protein. Some antibodies target an extracellular domain near the amino terminal for example, Santa Cruz Biotechnology, SC-1173 (amino acids 15–24), the transmembrane spanning regions of the receptor, intra- and extracellular domains between the transmembrane spanning domains, the third intracellular loop (amino acids 225–237) of the AT_1_ receptor (Chemicon), and the intracellular carboxy terminal domain. Several of these studies have used antibodies directed against the same carboxy terminal regions of the AT_1a_ (Abcam, AB18801), the AT_1a_ or the AT_1b_ (Advanced Targeting Systems, AB-N25AP, AB-N26AP, or AB-N27AP), and the AT_2_ receptors (Abcam, AB19134; Advanced Targeting Systems, AB-N28AP) that were used for generation of these antibodies.

Localization of AT_1_ receptor immunoreactivity in the brain was first done by Phillips et al., [145] using the 225–237 antibody directed to the third intracellular loop of the AT_1_ receptor. They showed extensive distribution of AT_1_ immunoreactivity in areas identified by receptor autoradiography to have Ang II receptors. Cardiovascular regulatory regions that were AT_1 _immunopositive included the PVN, OVLT, SFO, area postrema, NTS, RVLM, and nucleus ambiguous. AT_1_ immunopositive neurons were also present in the SON, and magnocellular division of the PVN, medial septal nucleus, LC, superior and inferior olivary nuclei, hypoglossal nucleus, ventral horn of the spinal cord and other regions not generally viewed as AT_1_ receptor targets of Ang II. Conversely, some areas reported to express Ang II receptor binding sites, for example, pyriform cortex, suprachiasmatic nucleus did not show AT_1_ immunoreactivity. They suggested that Ang II via AT_1_ receptors may have an expanded role in the CNS beyond that considered at that time. 

Other studies also report the presence of AT_1_ receptors in the SON and/or the magnocellular division of the PVN using either an amino terminal peptide fragment directed antibody, AB18801 and AB-N27AP [146, 147] and the antibody directed against the 225–237 fragment of the AT_1_ receptor [148, 149]. Of note, the number of cells in the magnocellular division of the PVN expressing AT_1 _receptor using AB18801 was dramatically increased in rats with induced heart failure [146]. Two other studies observed an increase in total PVN AT_1_ receptor immunoreactivity (Abcam unspecified). In the first study, PVN AT_1_ immunoreactivity was increased in a rat model of heart failure [150]. In the second study, PVN AT_1_ immunoreactivity was increased with chronic intravenous Ang II infusion that was only partially reversed by ICV losartan infusion [151].

Using an antibody against purified AT_2_ receptor protein, Reagan et al. [152] immunohistochemically localized AT_2_ receptor immunoreactivity in the rat brain. Regions reported to have AT_2_ receptor binding and/or mRNA that were immunopositive included the locus coeruleus and several thalamic nuclei. Other regions reported to be AT_2_ expressing included the amygdala and the Purkinje cell layer of the cerebellum. In addition, AT_2_ immunoreactivity was present in the magnocellular division of the PVN and SON which further confirms observations in our study. However, as noted above, this antibody did not label the adrenal [70].

A series of studies have used the AT_1a_ carboxy-terminal fragment-directed antibody to identify AT_1_ receptor immunoreactivity in the area postrema, NTS and RVLM at the electron microscopic level. AT_1a_ immunoreactivity was present in neuronal cell bodies, dendrites, axon terminals, perivascular glial processes of astrocytes, fibroblasts, and vascular endothelial cells in the area postrema and dorsomedial NTS [153]. This AT_1a_ immunoreactivity colocalized with the gp91^phox^ subunit of NADPH oxidase in neuronal cell bodies, dendrites, and putative vagal afferents in the medial NTS [154]. Dendritic processes of the medial NTS containing AT_1a_ immunoreactivity also were positive for tyrosine hydroxylase (TH) or adjacent to TH containing axons [155]. In the TH positive neurons of the RVLM, AT_1_ receptor expression was greater in female rats than in male rats [156], and this increase was associated with a higher estrogen state (proestrus versus diestrus) and increased plasma membrane expression of AT_1_ immunoreactivity [157]. This same group has used the AT_2_ fragment directed antibody (AB19134) to identify AT_2_ receptor immunoreactivity in the PVN and NTS at the electron microscopic level [158, 159]. These studies have co-localized AT_2_ immunoreactivity with neuronal nitric oxide synthase (nNOS) in neuronal cell bodies and dendrites in the medial NTS [159], and with vasopressin in neuronal cell bodies and dendrites in the PVN [158]. This latter observation contrasts with the studies of Lenkei et al. [142], who did not find AT_2_ receptor mRNA in the PVN. 

Extensive studies of AT_1_ and AT_2_ immunoreactivity in the RVLM and NTS in animal models of heart failure have been carried out by Gao, Zucker and colleagues using AT_1_ and AT_2_ antibodies, primarily SC-1173 and SC-9040 [160, 161]. AT_1_ receptors in the RVLM and NTS showed increased AT_1_ immunoreactivity, while AT_2_ receptors showed decreased immunoreactivity. Infusion of Ang II into the brain of rabbits to simulate a heart failure model increased AT_1_ receptor immunoreactivity in the RVLM [162]. Interestingly, viral transfection of AT_2_ receptors into the RVLM, which was documented with increased AT_2_ immunoreactivity, suppressed sympathetic activity in normal rats [163]. In a mouse model of hypertension, the RA mouse [164], immunoreactivity for AT_1_ (SC-1173) in the NTS and RVLM, was not shown to be up regulated [165].

AT_1_ (AB18801) and AT_2_ (AB19134) immunoreactivity in the substantia nigra (SN) colocalized with TH in neurons, GFAP in astrocytes and OX-6 and OX-42 in activated microglia [166–168]. Using different carboxy terminal directed AT_1_ and AT_2_ antibodies for Western blotting, it was shown that estrogen treatment of ovariectomized rats, which was protective against 6-hydroxydopamine induced neurotoxicity in the SN, decreased AT_1_ and increased AT_2_ expression in the SN [166]. Of note, no change in AT_1_ receptor mRNA was observed [166]. These researchers also observed AT_1_ and AT_2_ immunoreactivity (Santa Cruz, SC-579 and SC-9040) in dopaminergic neurons, astrocytes and microglia in both monkey and human SN [169].

The dorsomedial hypothalamus (DMH), a brain region that exhibits high AT_1_ receptor density [170], also displays AT_1_ immunoreactivity using the AB-N27AP [147]. This brain region is associated with the cardiovascular manifestations of panic disorder and direct administration of an AT_1 _receptor antagonist into the DMH blocks this component of the panic disorder in an animal model of panic disorder [147].

Giles et al., [67] using the 350–359 carboxy terminal peptide directed AT_1a_ antibody, observed strong AT_1_ receptor immunoreactivity in numerous brain regions including the SFO, OVLT, MnPO, the parvocellular division of the PVN, several other hypothalamic nuclei, and the NTS, corresponding well with radioligand binding and mRNA studies of the distribution of brain AT_1_ receptors.

### 4.6. Perspective on the Use of Antibodies for the Study of Angiotensin Receptors

 The ambiguity associated with studies of angiotensin receptors using different methods, whether by radioligand binding, receptor autoradiography, mRNA, local application of Ang II, electrophysiology, fos induction, or by immunoreactivity, necessitates considerable stringency in the analysis and interpretation of the data. Strengths of the immunohistochemical studies reported herein are as follows: (1) there is no known peptide sequence that closely mimics those used to generate these antibodies, (2) the antibodies were affinity purified to eliminate antibodies that did not recognize the antigenic peptide, (3) antibody binding is blocked by incubation with an excess of the antigenic peptide (preadsorption control), (4) Western blots indicate that the primary bands of labeled protein have molecular weights within the range of those previously observed for glycosylated, dimerized or chaperone protein linked angiotensin receptors [68, 171–175], and (5) the anatomical pattern of immunoreactivity correlates with radioligand binding for AT receptors [37, 59], agonist-induced c-fos expression [176], and the distribution of mRNA encoding the protein [44]. 

Weaknesses of this and other immunohistochemical approaches are as follows: (1) one cannot rule out the possibility that another protein could present an epitope similar to that recognized by these antibodies leading to a false positive, (2) there are posttranslational modifications of the receptor proteins that may mask the antigenic sites that they recognize, for example, phosphorylation of serine residues in Ang II receptors by a variety of protein kinases. The C-terminal domains chosen for generation of these antibodies contain several serines which when phosphorylated may mask the epitopes for the antibodies. AT_1 _receptors are phosphorylated by G protein receptor kinase GRK2 (formerly known as *β* adrenergic receptor kinase, BARK1) leading to *β*-arrestin binding to the intracellular domain of the AT_1_ receptors which may also mask the epitopes [177]. An additional post-translational modification is proteolytic cleavage of the receptor into smaller fragments following internalization. Cook et al. [178] demonstrated formation of a 54 amino acid carboxy terminal fragment of the rat AT_1a_ receptor that translocated to the nucleus and induced apoptosis in a variety of cell types. Thus it is possible that the immunoreactivity observed herein is not that of the full length receptor. (3) Receptors undergo protein-protein interactions such as receptor dimerization or interactions with chaperone proteins which have the potential to mask the antigenic site on the receptor; (4) inability to document the loss of immunological reactivity in an animal in which the receptor protein has been eliminated, for example, receptor knockouts. A recent publication [179] using Western blotting and immunofluorescence has challenged the specificity of 6 commercially available AT_1_ receptor antibodies, including one previously questioned by Adams et al., [180] based upon the presence of immunoreactive material in mice in which the AT_1a_ receptor is disrupted. The specificity of 3 AT_1_ receptor antibodies, Alomone Labs #AAR-011, Santa Cruz sc-1173, and Abcam 18801, has also been challenged based upon expression of immunoreactivity in AT_1a_ and AT_1b_ knockout mice [181]. A generalized challenge to the ability of antibodies to selectively recognize G protein-coupled receptors (GPCR) based on apparent nonspecificity of 49 GPCR antibodies to 19 different GPCRs (the AT_1_ and AT_2_ receptors were not among the 19 GPCRs) has called into question the validity of immunological identification of GPCRs [182]. However, Xue et al., [183] using the same antibody as Adams et al., [180] demonstrated knockdown of AT_1_ receptor immunoreactivity in the PVN. Of note, the AT_1a_ gene disruption [184] does not eliminate the carboxy terminal coding domain of the receptor that includes the peptide sequences used to generate several of those antibodies. If this portion of the receptor is still expressed it could explain the residual presence of AT_1a_ immunoreactive material in these knockout mice. However, the amino terminal sequence used to generate SC-1173 (amino acids 15–24) is in the deleted part; thus, it remains questionable whether the siRNA knockdown in the rat brain or the knockout of the mouse AT_1a_ receptor gives the correct information regarding the specificity of this and other AT_1_ receptor antibodies. 

One approach to resolve this question is to determine the identity of the protein in the band that the AT_1_ receptor antibodies recognize in both wild-type and AT_1_ receptor knockout mice. This has the potential to either (1) validate the immunological identification of AT_1_ receptor protein thereby calling into question the efficacy of the AT_1_ receptor knockout technology, (2) to discover a heretofore unknown subtype of the AT_1_ receptor with an mRNA sequence that somehow evaded recognition by homology cloning approaches, (3) to identify (a) non-AT_1 _protein(s) that colocalize(s) with AT_1_ receptors and display (a) sufficiently similar epitope(s) as to be recognized by a variety of AT_1_ receptor antibodies, (4) to discover (a) proteins with no relationship to AT_1_ receptors that coincidentally express the same epitope(s) as the AT_1 _receptor antibodies, or (5) to discover (a) novel protein(s) that has/have not yet been identified.

Until such questions are definitively answered, immunohistochemical studies, despite their known and potential limitations, can complement other types of analyses, which are also subject to a variety of differing limitations. 

In conclusion, antibodies that can differentiate the 3 different angiotensin II receptor subtypes in the rat were used to immunohistochemically label angiotensin II receptor subtype-like immunoreactivity in the rat adrenal, pituitary, and brain. The pattern of staining corroborates mRNA, radioligand binding, and functional studies of adrenal and anterior pituitary angiotensin receptors. This indicates that AT_1a_ and AT_2_ receptor subtypes occur in the zona glomerulosa and medulla of normal rats, the AT_1b_ subtype occurs only in the zona glomerulosa of normal rats while the AT_1b_ is the subtype predominantly expressed in the anterior pituitary. The localization of Ang II receptor immunoreactivity in the brain is in large part consistent with radioligand binding, mRNA, Ang II-induced fos expression, and functional studies; however, differences between these immunoreactivity observations and observations obtained from some other techniques are yet to be resolved.

## Figures and Tables

**Table 1:** Tabulated summary of comparative regional and cellular distribution of Ang II receptor-immunoreactivity in rat brain and pituitary.

Region	AT_1a_	AT_1b _	AT_2_
Neocortex			
Lamina I-II	ND	0^+^/5*	1^+^/1*
Lamina III-IV	ND	4^+^/5*	0^+^/4*
Lamina V-VI	ND	4^+^/4*	3^+^/3*
Basal (Anterior) Forebrain			
Entorhinal cortex	2^+^/0*	0^+^/0*	5^+^/1*
Hippocampus			
CA1	1^+^/0*	ND	4^+^/5*
CA3	1^+^/0*	ND	5^+^/5*
Dentate Gyrus	ND	ND	2^+^/2*
Central Amygdala	1^+^/0*	ND	4^+^/3*
Caudate nucleus	3^+^/1*	1^+^/0*	5^+^/3*
Thalamus			
Medial Dorsal Thalamus	0^+^/0*	0^+^/0*	5^+^/2*
Periventricular nucleus of the thalamus	0^+^/0*	0^+^/0*	3^+^/5*
Medial Habenula	1^+^/0*	0^+^/0*	5^+^/3*
Lateral Habenula	0^+^/0*	0^+^/0*	0^+^/0*
Septal Area			
Dorsal Median preoptic nucleus	2^+^/3*	3^+^/4*	4^+^/5*
Medial Septum	0^+^/0*	0^+^/0*	0^+^/2*
Lateral Septum	2^+^/1*	1^+^/1*	2^+^/0*
Hypothalamus			
Anterior Hypothalamic Area	2^+^/3*	1^+^/1*	3^+^/4*
Lateral Hypothalamic Area	3^+^/2^+^	4^+^/5*	3^+^/0*
Paraventricular nucleus	4^+^/0*	5^+^/5*	5^+^/3*
Periventricular area	3^+^/0*	2^+^/5	5^+^/0*
Suprachiasmatic nucleus	1^+^/0*	0^+^/0*	2^+^/5*
Arcuate nucleus	5^+^/4*	5^+^/5*	4^+^/0*
Circumventricular Organs/Pituitary			
Median Eminence	0^+^/2*	0^+^/3*	0^+^/4^+^
Subfornical Organ	4^+^/5*	0^+^/2*	3^+^/5*
Area Postrema	3^+^/0*	4^+^/4*	3^+^/4*
Posterior Pituitary (pars nervosa)	0^+^/3*	0^+^/2*	ND
Anterior Pituitary (pars distalis)	0^+^/0*	5^+^/0*	5^+^/0*
Stellate cells	0^+^/0*	5^+^/0*	1^+^/0*
Ovoid cells	0^+^/0*	1^+^/0*	4^+^/0*
Cerebellum			
Purkinje Cells	0^+^/0*	5^+^/5*	ND
Hindbrain			
RVLM	4^+^/2*	2^+^/2*	ND
NTS	3^+^/4*	5^+^/2*	3^+^/4*

Key: ^+^refers to neuronal cell bodies/*refers to fibers. Scored on a scale from 0 to 5. ND is not determined. No AT_1a_ immunoreactivity was observed in glia. AT_1b_ and AT_2_ immunoreactivity were observed in glia.

## References

[1] Laragh JH, Angers M, Kelly WG, Lieberman S (1960). Hypotensive agents and pressor substances. The effect of epinephrine, norepinephrine, angiotensin II, and others on the secretory rate of aldosterone in man. *The Journal of the American Medical Association*.

[2] Davis JO, Hartroft PM, Titus EO, Carpenter CCJ, Ayers CR, Spiegel HE (1962). The role of the renin-angiotensin system in the control of aldosterone secretion. *The Journal of Clinical Investigation*.

[3] Feldberg W, Lewis GP (1964). The action of peptides on the adrenal medulla. Release of adrenaline by bradykinin and angiotensin. *The Journal of physiology*.

[4] Ganten D, Minnich JL, Granger P (1971). Angiotensin-forming enzyme in brain tissue. *Science*.

[5] Stornetta RL, Hawelu-Johnson CL, Guyenet PG, Lynch KR (1988). Astrocytes synthesize angiotensinogen in brain. *Science*.

[6] Lind RW, Swanson LW, Ganten D (1985). Organization of angiotensin II immunoreactive cells and fibers in the rat central nervous system. An immunohistochemical study. *Neuroendocrinology*.

[7] Booth DA (1968). Mechanism of action of norepinephrine in eliciting an eating response on injection into the rat hypothalamus. *Journal of Pharmacology and Experimental Therapeutics*.

[8] Reid IA, Ramsay DJ (1975). The effects of intracerebroventricular administration of renin on drinking and blood pressure. *Endocrinology*.

[9] Severs WB, Daniels-Severs AE (1973). Effects of angiotensin on the central nervous system. *Pharmacological Reviews*.

[10] Phillips MI, Sumners C (1998). Angiotensin II in central nervous system physiology. *Regulatory Peptides*.

[11] Wright JW, Harding JW (2013). The brain renin-angiotensin system: a diversity of functions and implications for CNS diseases. *Pflügers Archiv*.

[12] Ganong WF (1993). Blood, pituitary, and brain renin-angiotensin systems and regulation of secretion of anterior pituitary gland. *Frontiers in Neuroendocrinology*.

[13] Vila-Porcile E, Corvol P (1998). Angiotensinogen, prorenin, and renin are co-localized in the secretory granules of all glandular cells of the rat anterior pituitary: an immunoultrastructural study. *Journal of Histochemistry and Cytochemistry*.

[14] Chiu AT, Herblin WF, McCall DE (1989). Identification of angiotensin II receptor subtypes. *Biochemical and Biophysical Research Communications*.

[15] Whitebread S, Mele M, Kamber B, De Gasparo M (1989). Preliminary biochemical characterization of two angiotensin II receptor subtypes. *Biochemical and Biophysical Research Communications*.

[16] Murphy TJ, Alexander RW, Griendling KK, Runge MS, Bernstein KE (1991). Isolation of a cDNA encoding the vascular type-1 angiotensin II receptor. *Nature*.

[17] Sasaki K, Yamano Y, Bardhan S (1991). Cloning and expression of a complementary DNA encoding a bovine adrenal angiotensin II type-1 receptor. *Nature*.

[18] Kambayashi Y, Bardhan S, Takahashi K (1993). Molecular cloning of a novel angiotensin II receptor isoform involved in phosphotyrosine phosphatase inhibition. *Journal of Biological Chemistry*.

[19] Mukoyama M, Nakajima M, Horiuchi M, Sasamura H, Pratt RE, Dzau VJ (1993). Expression cloning of type 2 angiotensin II receptor reveals a unique class of seven-transmembrane receptors. *Journal of Biological Chemistry*.

[20] Tofovic SP, Pong AS, Jackson EK (1991). Effects of angiotensin subtype 1 and subtype 2 receptor antagonists in normotensive versus hypertensive rats. *Hypertension*.

[21] Hano T, Mizukoshi M, Baba A, Nakamura N, Nishio I (1994). Angiotensin II subtype 1 receptor modulates epinephrine release from isolated rat adrenal gland. *Blood Pressure, Supplement*.

[22] Kirby RF, Thunhorst RL, Johnson AK (1992). Effects of a non-peptide angiotensin receptor antagonist on drinking and blood pressure responses to centrally administered angiotensins in the rat. *Brain Research*.

[23] Hogarty DC, Speakman EA, Puig V, Phillips MI (1992). The role of angiotensin, AT_1_ and AT_2_ receptors in the pressor, drinking and vasopressin responses to central angiotensin. *Brain Research*.

[24] Lazartigues E, Sinnayah P, Augoyard G, Gharib C, Johnson AK, Davisson RL (2008). Enhanced water and salt intake in transgenic mice with brain-restricted overexpression of angiotensin (AT_1_) receptors. *American Journal of Physiology*.

[25] Colombari DSA, Menani JV, Johnson AK (1996). Forebrain angiotensin type 1 receptors and parabrachial serotonin in the control of NaCl and water intake. *American Journal of Physiology*.

[26] Camargo LAA, Saad WA, Simões S, Santos TAB, Abrão Saad W (2002). Interaction between paraventricular nucleus and septal area in the control of physiological responses induced by angiotensin II. *Brazilian Journal of Medical and Biological Research*.

[27] Rowe BP, Grove KL, Saylor DL, Speth RC (1990). Angiotensin II receptor subtypes in the rat brain. *European Journal of Pharmacology*.

[28] Tsutsumi K, Saavedra JM (1991). Quantitative autoradiography reveals different angiotensin II receptor subtypes in selected rat brain nuclei. *Journal of Neurochemistry*.

[29] Song K, Allen AM, Paxinos G, Mendelsohn FAO (1991). Angiotensin II receptor subtypes in rat brain. *Clinical and Experimental Pharmacology and Physiology*.

[30] Nitabach MN, Schulkin J, Epstein AN (1989). The medial amygdala is part of a mineralocorticoid-sensitive circuit controlling NaCl intake in the rat. *Behavioural Brain Research*.

[31] Kakar SS, Riel KK, Neill JD (1992). Differential expression of angiotensin II receptor subtype mRNAs (AT-1A and AT-1B) in the brain. *Biochemical and Biophysical Research Communications*.

[32] Iwai N, Inagami T (1992). Identification of two subtypes in the rat type I angiotensin II receptor. *FEBS Letters*.

[33] Elton TS, Stephan CC, Taylor GR (1992). Isolation of two distinct type I angiotensin II receptor genes. *Biochemical and Biophysical Research Communications*.

[34] Chiu AT, Dunscomb J, Kosierowski J (1993). The ligand binding signatures of the rat AT_1a_, AT_1b_ and the human AT_1_ receptors are essentially identical. *Biochemical and Biophysical Research Communications*.

[35] Sandberg K, Ji H, Clark AJL, Shapira H, Catt KJ (1992). Cloning and expression of a novel angiotensin II receptor subtype. *Journal of Biological Chemistry*.

[36] Tian Y, Baukal AJ, Sandberg K, Bernstein KE, Balla T, Catt KJ (1996). Properties of AT_1a_ and AT_1b_ angiotensin receptors expressed in adrenocortical Y-1 cells. *American Journal of Physiology*.

[37] Speth RC (2003). Sarcosine1,glycine8 angiotensin II is an AT 1 angiotensin II receptor subtype selective antagonist. *Regulatory Peptides*.

[38] Kakar SS, Sellers JC, Devor DC, Musgrove LC, Neill JD (1992). Angiotensin II type-1 receptor subtype cDNAs: differential tissue expression and hormonal regulation. *Biochemical and Biophysical Research Communications*.

[39] Jöhren O, Golsch C, Dendorfer A, Qadri F, Häuser W, Dominiak P (2003). Differential expression of AT_1_ receptors in the pituitary and adrenal gland of SHR and WKY. *Hypertension*.

[40] Burson JM, Aguilera G, Gross KW, Sigmund CD (1994). Differential expression of angiotensin receptor 1A and 1B in mouse. *American Journal of Physiology*.

[41] Lenkei Z, Corvol P, Llorens-Cortes C (1995). The angiotensin receptor subtype AT_1a_ predominates in rat forebrain areas involved in blood pressure, body fluid homeostasis and neuroendocrine control. *Molecular Brain Research*.

[42] Chen Y, Morris M (2001). Differentiation of brain angiotensin type 1a and 1b receptor mRNAs a specific effect of dehydration. *Hypertension*.

[43] Krishnamurthi K, Verbalis JG, Zheng W, Wu Z, Clerch LB, Sandberg K (1999). Estrogen regulates angiotensin AT_1_ receptor expression via cytosolic proteins that bind to the 5’ leader sequence of the receptor mRNA. *Endocrinology*.

[44] Gasc JM, Shanmugam S, Sibony M, Corvol P (1994). Tissue-specific expression of type 1 angiotensin II receptor subtypes: an in situ hybridization study. *Hypertension*.

[45] Johren O, Inagami T, Saavedra JM (1995). AT_1a_, AT_1b_, and AT_2_ angiotensin II receptor subtype gene expression in rat brain. *NeuroReport*.

[46] Jezova M, Armando I, Bregonzio C (2003). Angiotensin II AT_1_ and AT_2_ receptors contribute to maintain basal adrenomedullary norepinephrine synthesis and tyrosine hydroxylase transcription. *Endocrinology*.

[47] Lenkei Z, Palkovits M, Corvol P, Llorens-Cortès C (1997). Expression of angiotensin type-1 (AT_1_) and type-2 (AT_2_) receptor mRNAs in the adult rat brain: a functional neuroanatomical review. *Frontiers in Neuroendocrinology*.

[48] Hopp TP, Woods KR (1981). Prediction of protein antigenic determinants from amino acid sequences. *Proceedings of the National Academy of Sciences of the United States of America*.

[49] Kitami Y, Okura T, Marumoto K, Wakamiya R, Hiwada K (1992). Differential gene expression and regulation of type-1 angiotensin II receptor subtypes in the rat. *Biochemical and Biophysical Research Communications*.

[50] Chiu AT, Dunscomb JH, McCall DE, Benfield P, Baubonis W, Sauer B (1993). Characterization of angiotensin AT_1a_ receptor isoform by its ligand binding signature. *Regulatory Peptides*.

[51] Jo H, Yang EK, Lee WJ, Park KY, Kim HJ, Park JS (1996). Gene expression of central and peripheral renin-angiotensin system components upon dietary sodium intake in rats. *Regulatory Peptides*.

[52] Llorens-Cortes C, Greenberg B, Huang H, Corvol P (1994). Tissular expression and regulation of type 1 angiotensin II receptor subtypes by quantitative reverse transcriptase-polymerase chain reaction analysis. *Hypertension*.

[53] Qiu J, Nelson SH, Speth RC, Wang DH (1999). Regulation of adrenal angiotensin receptor subtypes: a possible mechanism for sympathectomy-induced adrenal hypertrophy. *Journal of Hypertension*.

[54] Iwai N, Inagami T, Ohmichi N, Nakamura Y, Saeki Y, Kinoshita M (1992). Differential regulation of rat AT_1a_ and AT_1b_ receptor mRNA. *Biochemical and Biophysical Research Communications*.

[55] Wang DH, Du Y, Zhao H, Granger JP, Speth RC, Dipette DJ (1997). Regulation of Angiotensin type 1 receptor and its gene expression: role in renal growth. *Journal of the American Society of Nephrology*.

[56] Martin MM, Lee EJ, Buckenberger JA, Schmittgen TD, Elton TS (2006). MicroRNA-155 regulates human angiotensin II type 1 receptor expression in fibroblast. *Journal of Biological Chemistry*.

[57] Naruse M, Tanabe A, Sugaya T (1998). Deferential roles of angiotensin receptor subtypes in adrenocortical function in mice. *Life Sciences*.

[58] Song K, Zhuo J, Allen AM, Paxinos G, Mendelsohn FAO (1991). Angiotensin II receptor subtypes in rat brain and peripheral tissues. *Cardiology*.

[59] Israel A, Plunkett LM, Saavedra JM (1985). Quantitative autoradiographic characterization of receptors for angiotensin II and other neuropeptides in individual brain nuclei and peripheral tissues from single rats. *Cellular and Molecular Neurobiology*.

[60] Healy DP, Maciejewski AR, Printz MP (1985). Autoradiographic localization of [125I]-angiotensin II binding sites in the rat adrenal gland. *Endocrinology*.

[61] Birukov KG, Lehoux S, Birukova AA, Merval R, Tkachuk VA, Tedgui A (1997). Increased pressure induces sustained protein kinase C-independent herbimycin A-sensitive activation of extracellular signal-related kinase 1/2 in the rabbit aorta in organ culture. *Circulation Research*.

[62] Wakamiya R, Kohara K, Hiwada K (1994). Gene expression of the type-1 angiotensin II receptor in rat adrenal gland. *Blood Pressure*.

[63] Harada K, Matsuoka H, Fujimoto N (2010). Localization of type-2 angiotensin II receptor in adrenal gland. *Journal of Histochemistry and Cytochemistry*.

[64] Peters B, Clausmeyer S, Teubner P (2001). Changes of AT_2_ receptor levels in the rat adrenal cortex and medulla induced by bilateral nephrectomy and its modulation by circulating ANG II. *Journal of Histochemistry and Cytochemistry*.

[65] Paxton WG, Runge M, Horaist C, Cohen C, Alexander RW, Bernstein KE (1993). Immunohistochemical localization of rat angiotensin II AT_1_ receptor. *American Journal of Physiology*.

[66] Lehoux JG, Bird IM, Briere N, Martel D, Ducharme L (1997). Influence of dietary sodium restriction on angiotensin II receptors in rat adrenals. *Endocrinology*.

[67] Giles ME, Fernley RT, Nakamura Y (1999). Characterization of a specific antibody to the rat angiotensin II AT_1_ receptor. *Journal of Histochemistry and Cytochemistry*.

[68] Frei N, Weissenberger J, Beck-Sickinger AG, Höfliger M, Weis J, Imboden H (2001). Immunocytochemical localization of angiotensin II receptor subtypes and angiotensin II with monoclonal antibodies in the rat adrenal gland. *Regulatory Peptides*.

[69] Yiu AKL, Wong PF, Yeung SY, Lam SM, Luk SKS, Cheung WT (1997). Immunohistochemical localization of type-II (AT_2_) angiotensin receptors with a polyclonal antibody against a peptide from the C-terminal tail. *Regulatory Peptides*.

[70] Reagan LP, Sakai RR, Fluharty SJ (1996). Immunological analysis of angiotensin AT_2_ receptors in peripheral tissues of neonatal and adult rats. *Regulatory Peptides*.

[71] Ferguson SSG (2001). Evolving concepts in G protein-coupled receptor endocytosis: the role in receptor desensitization and signaling. *Pharmacological Reviews*.

[72] Hunyady L, Baukal AJ, Gáborik Z (2002). Differential PI 3-kinase dependence of early and late phases of recycling of the internalized AT_1_ angiotensin receptor. *Journal of Cell Biology*.

[73] Kumar R, Singh VP, Baker KM (2007). The intracellular renin-angiotensin system: a new paradigm. *Trends in Endocrinology and Metabolism*.

[74] Boivin B, Vaniotis G, Allen BG, Hébert TE (2008). G protein-coupled receptors in and on the cell nucleus: a new signaling paradigm?. *Journal of Receptors and Signal Transduction*.

[75] Robertson AL, Khairallah PA (1971). Angiotensin II: rapid localization in nuclei of smooth and cardiac muscle. *Science*.

[76] Re R, Parab M (1984). Effect of angiotensin II on RNA synthesis by isolated nuclei. *Life Sciences*.

[77] Chiu AT, McCall DE, Nguyen TT (1989). Discrimination of angiotensin II receptor subtypes by dithiothreitol. *European Journal of Pharmacology*.

[78] Eggena P, Zhu JH, Clegg K, Barrett JD (1993). Nuclear angiotensin receptors induce transcription of renin and angiotensinogen mRNA. *Hypertension*.

[79] Eggena P, Zhu JH, Sereevinyayut S (1996). Hepatic angiotensin II nuclear receptors and transcription of growth-related factors. *Journal of Hypertension*.

[80] Re RN, Vizard DL, Brown J, Bryan SE (1984). Angiotensin II receptors in chromatin fragments generated by micrococcal nuclease. *Biochemical and Biophysical Research Communications*.

[81] Booz GW, Conrad KM, Hess AL, Singer HA, Baker KM (1992). Angiotensin-II-binding sites on hepatocyte nuclei. *Endocrinology*.

[82] Tang SS, Rogg H, Schumacher R, Dzau VJ (1992). Characterization of nuclear angiotensin-II-binding sites in rat liver and comparison with plasma membrane receptors. *Endocrinology*.

[83] Licea H, Walters MR, Navar LG (2002). Renal nuclear angiotensin II receptors in normal and hypertensive rats. *Acta physiologica Hungarica*.

[84] Tadevosyan A, Maguy A, Villeneuve LR (2010). Nuclear-delimited angiotensin receptor-mediated signaling regulates cardiomyocyte gene expression. *Journal of Biological Chemistry*.

[85] Chen R, Mukhin YV, Garnovskaya MN (2000). A functional angiotensin II receptor-GFP fusion protein: evidence for agonist-dependent nuclear translocation. *American Journal of Physiology*.

[86] Morinelli TA, Raymond JR, Baldys A (2007). Identification of a putative nuclear localization sequence within ANG II AT_1a_ receptor associated with nuclear activation. *American Journal of Physiology*.

[87] Lu D, Yang H, Shaw G, Raizada MK (1998). Angiotensin II-induced nuclear targeting of the angiotensin type 1 (AT_1_) receptor in brain neurons. *Endocrinology*.

[88] Lee DK, Lança AJ, Cheng R (2004). Agonist-independent nuclear localization of the apelin, angiotensin AT 1, and bradykinin B2 receptors. *Journal of Biological Chemistry*.

[89] Goodfriend TL, Peach MJ (1975). Angiotensin III: (Des aspartic acid) angiotensin II. Evidence and speculation for its role as an important agonist in the renin angiotensin system. *Circulation Research*.

[90] Shanmugam S, Lenkei ZG, Gasc JMR, Corvol PL, Llorens-Cortes CM (1995). Ontogeny of angiotensin II type 2 (AT_2_) receptor mRNA in the rat. *Kidney International*.

[91] Israel A, Correa FMA, Niwa M, Saavedra JM (1984). Quantitative measurement of angiotensin II (A II) receptors in discrete regions of rat brain, pituitary and adrenal gland by autoradiography. *Clinical and Experimental Hypertension A*.

[92] Speth RC, Wamsley JK, Gehlert DR (1985). Angiotensin II receptor localization in the canine CNS. *Brain Research*.

[93] Gehlert DR, Speth RC, Wamsley JK (1986). Distribution of [125I]angiotensin II binding sites in the rat brain: a quantitative autoradiographic study. *Neuroscience*.

[94] Tsutsumi K, Saavedra JM (1991). Angiotensin-II receptor subtypes in median eminence and basal forebrain areas involved in regulation of pituitary function. *Endocrinology*.

[95] Saavedra JM (1992). Brain and pituitary angiotensin. *Endocrine Reviews*.

[96] Pawlikowski M (2006). Immunohistochemical detection of angiotensin receptors AT_1_ and AT_2_ in normal rat pituitary gland, estrogen-induced rat pituitary tumor and human pituitary adenomas. *Folia Histochemica et Cytobiologica*.

[97] Shelat SG, Reagan LP, King JL, Fluharty SJ, Flanagan-Cato LM (1998). Analysis of angiotensin type 2 receptors in vasopressinergic neurons and pituitary in the rat. *Regulatory Peptides*.

[98] Lenkei Z, Nuyt AM, Grouselle D, Corvol P, Llorens-Cortès C (1999). Identification of endocrine cell populations expressing the AT_1b_ subtype of angiotensin II receptors in the anterior pituitary. *Endocrinology*.

[99] Sanvitto GL, Jöhren O, Häuser W, Saavedra JM (1997). Water deprivation upregulates ANG II AT_1_ binding and mRNA in rat subfornical organ and anterior pituitary. *American Journal of Physiology*.

[100] Moreau C, Rasolojanahary R, Zamora AJ, Enjalbert A, Kordon C, Llorens-Cortes C (1997). Expression of angiotensin II receptor subtypes AT_1a_ and AT_1b_ in enriched fractions of dispersed rat pituitary cells. *Neuroendocrinology*.

[101] Bonjour JP, Malvin RL (1970). Stimulation of ADH release by the renin-angiotensin system. *The American journal of physiology*.

[102] Brooks VL, Keil LC, Reid IA (1986). Role of the renin-angiotensin system in the control of vasopressin secretion in conscious dogs. *Circulation Research*.

[103] Zimmerman BG (1962). Effect of acute sympathectomy on responses to angiotensin and norepinephrine. *Circulation research*.

[104] Zimmerman BG (1981). Adrenergic facilitation by angiotensin: does it serve a physiological function?. *Clinical Science*.

[105] Aguilera G, Hyde CL, Catt KJ (1982). Angiotensin II receptors and prolactin release in pituitary lactotrophs. *Endocrinology*.

[106] Steele MK, McCann SM, Negro-Vilar A (1982). Modulation by dopamine and estradiol of the central effects of angiotensin II on anterior pituitary hormone release. *Endocrinology*.

[107] Bluet-Pajot MT, Epelbaum J, Gourdji D, Hammond C, Kordon C (1998). Hypothalamic and hypophyseal regulation of growth hormone secretion. *Cellular and Molecular Neurobiology*.

[108] Spinedi E, Herrera L, Chisari A (1988). Angiotensin II (AII) and adrenocorticotropin release: modulation by estradiol of the AII biological activity and binding characteristics in anterior pituitary dispersed cells. *Endocrinology*.

[109] Seltzer A, Pinto JEB, Viglione PN (1992). Estrogens regulate angiotensin-converting enzyme and angiotensin receptors in female rat anterior pituitary. *Neuroendocrinology*.

[110] Johren O, Sanvitto GL, Egidy G, Saavedra JM (1997). Angiotensin II AT_1a_ receptor mRNA expression is induced by estrogen-progesterone in dopaminergic neurons of the female rat arcuate nucleus. *Journal of Neuroscience*.

[111] Inagami T, Eguchi S, Numaguchi K (1999). Cross-talk between angiotensin II receptors and the tyrosine kinases and phosphatases. *Journal of the American Society of Nephrology*.

[112] Gelband CH, Zhu M, Lu D (1997). Functional interactions between neuronal AT_1_ and AT_2_ receptors. *Endocrinology*.

[113] Sohn HY, Raff U, Hoffmann A (2000). Differential role of angiotensin II receptor subtypes on endothelial superoxide formation. *British Journal of Pharmacology*.

[114] Sumners C, Tang W, Paulding W, Raizada MK (1994). Peptide receptors in astroglia: focus on angiotensin II and atrial natriuretic peptide. *GLIA*.

[115] Saavedra JM (1999). Emerging features of brain angiotensin receptors. *Regulatory Peptides*.

[116] Füchtbauer L, Groth-Rasmussen M, Holm TH (2011). Angiotensin II Type 1 receptor (AT_1_) signaling in astrocytes regulates synaptic degeneration-induced leukocyte entry to the central nervous system. *Brain, Behavior, and Immunity*.

[117] Downie LE, Vessey K, Miller A (2009). Neuronal and glial cell expression of angiotensin II type 1 (AT_1_) and type 2 (AT_2_) receptors in the rat retina. *Neuroscience*.

[118] Benicky J, Sánchez-Lemus E, Honda M (2011). Angiotensin II AT_1_ receptor blockade ameliorates brain inflammation. *Neuropsychopharmacology*.

[119] Shi P, Raizada MK, Sumners C (2010). Brain cytokines as neuromodulators in cardiovascular control. *Clinical and Experimental Pharmacology and Physiology*.

[120] Shi P, Diez-Freire C, Jun JY (2010). Brain microglial cytokines in neurogenic hypertension. *Hypertension*.

[121] Bickerton RK, Buckley JP (1961). Evidence for a central mechanism in angiotensin induced hypertension. *Proceedings of the Society for Experimental Biology and Medicine*.

[122] Glossmann H, Baukal AJ, Catt KJ (1974). Properties of angiotensin II receptors in the bovine and rat adrenal cortex. *Journal of Biological Chemistry*.

[123] Bennett JP, Snyder SH (1976). Angiotensin II binding to mammalian brain membranes. *Journal of Biological Chemistry*.

[124] Van Houten M, Schiffrin EL, Mann JFE (1980). Radioautographic localization of specific binding sites for blood-borne angiotensin II in the rat brain. *Brain Research*.

[125] Mendelsohn FAO, Quirion R, Saavedra JM, Aguilera G, Catt KJ (1984). Autoradiographic localization of angiotensin II receptors in rat brain. *Proceedings of the National Academy of Sciences of the United States of America*.

[126] Rowe BP, Saylor DL, Speth RC (1992). Analysis of angiotensin II receptor subtypes in individual rat brain nuclei. *Neuroendocrinology*.

[127] Epstein AN, Fitzsimons JT, Rolls BJ (1970). Drinking induced by injection of angiotensin into the rain of the rat. *Journal of Physiology*.

[128] Johnson AK, Epstein AN (1975). The cerebral ventricles as the avenue for the dipsogenic action of intracranial angiotensin. *Brain Research*.

[129] Ferguson AV, Washburn DLS, Latchford KJ (2001). Hormonal and neurotransmitter roles for angiotensin in the regulation of central autonomic function. *Proceedings of the Society for Experimental Biology and Medicine*.

[130] Fontes MA, Baltatu O, Caligiorne SM (2000). Angiotensin peptides acting at rostral ventrolateral medulla contribute to hypertension of TGR(mREN2)27 rats. *Physiol Genomics*.

[131] Casto R, Phillips MI (1984). Cardiovascular actions of microinjections of angiotensin II in the brain stem of rats. *American Journal of Physiology*.

[132] D’Amico M, Di FC, Berrino L, Rossi F (1997). AT_1_ receptors mediate pressor responses induced by angiotensin II in the periaqueductal gray area of rats. *Life Sciences*.

[133] D’Amico M, Di FC, Rossi F, Warner TD (1998). Role of AT_2_ receptors in the cardiovascular events following microinjection of angiotensin II into the superior colliculus of anaesthetised rats. *Naunyn-Schmiedeberg’s Archives of Pharmacology*.

[134] McKinley MJ, Badoer E, Vivas L, Oldfield BJ (1995). Comparison of c-fos expression in the lamina terminalis of conscious rats after intravenous or intracerebroventricular angiotensin. *Brain Research Bulletin*.

[135] Nagatomo T, Inenaga K, Yamashita H (1995). Transient outward current in adult rat supraoptic neurones with slice patch-clamp technique: inhibition by angiotensin II. *Journal of Physiology*.

[136] Bourque CW, Voisin DL, Chakfe Y (2002). Stretch-inactivated cation channels: cellular targets for modulation of osmosensitivity in supraoptic neurons. *Progress in Brain Research*.

[137] Lenkei Z, Corvol P, Llorens-Cortes C (1995). Comparative expression of vasopressin and angiotensin type-1 receptor mRNA in rat hypothalamic nuclei: a double in situ hybridization study. *Molecular Brain Research*.

[138] Jöhren O, Saavedra JM (1996). Expression of AT_1a_ and AT_1b_ angiotensin II receptor messenger RNA in forebrain of 2-wk-old rats. *American Journal of Physiology*.

[139] Gehlert DR, Gackenheimer SL, Schober DA (1991). Autoradiographic localization of subtypes of angiotensin II antagonist binding in the rat brain. *Neuroscience*.

[140] Tsutsumi K, Saavedra JM (1991). Characterization and development of angiotensin II receptor subtypes (AT_1_ and AT_2_) in rat brain. *American Journal of Physiology*.

[141] Jöhren O, Inagami T, Saavedra JM (1996). Localization of AT_2_ angiotensin II receptor gene expression in rat brain by in situ hybridization histochemistry. *Molecular Brain Research*.

[142] Lenkei Z, Palkovits M, Corvol P, Llorens-Cortes C (1996). Distribution of angiotensin II type-2 receptor (AT_2_) mRNA expression in the adult rat brain. *Journal of Comparative Neurology*.

[143] Nunes FC, Braga VA (2011). Chronic angiotensin II infusion modulates angiotensin II type I receptor expression in the subfornical organ and the rostral ventrolateral medulla in hypertensive rats. *Journal of the Renin-Angiotensin-Aldosterone System*.

[144] Reagan LP, Theveniau M, Yang XD (1993). Development of polyclonal antibodies against angiotensin type 2 receptors. *Proceedings of the National Academy of Sciences of the United States of America*.

[145] Phillips MI, Shen L, Richards EM, Raizada MK (1993). Immunohistochemical mapping of angiotensin AT_1_ receptors in the brain. *Regulatory Peptides*.

[146] Wei SG, Yu Y, Zhang ZH, Weiss RM, Felder RB (2008). Mitogen-activated protein kinases mediate upregulation of hypothalamic angiotensin II type 1 receptors in heart failure rats. *Hypertension*.

[147] Shekhar A, Johnson PL, Sajdyk TJ (2006). Angiotensin-II is a putative neurotransmitter in lactate-induced panic-like responses in rats with disruption of GABAergic inhibition in the dorsomedial hypothalamus. *Journal of Neuroscience*.

[148] Moellenhoff E, Blume A, Culman J (2001). Effect of repetitive icv injections of ANG II on c-Fos and AT_1_-receptor expression in the rat brain. *American Journal of Physiology*.

[149] Rowland NE, Li BH, Fregly MJ, Smith GC (1995). Fos induced in brain of spontaneously hypertensive rats by angiotensin II and co-localization with AT-1 receptors. *Brain Research*.

[150] Kang YM, Ma Y, Elks C, Zheng JP, Yang ZM, Francis J (2008). Cross-talk between cytokines and renin-angiotensin in hypothalamic paraventricular nucleus in heart failure: role of nuclear factor-*κ*B. *Cardiovascular Research*.

[151] Kang YM, Ma Y, Zheng JP (2009). Brain nuclear factor-kappa B activation contributes to neurohumoral excitation in angiotensin II-induced hypertension. *Cardiovascular Research*.

[152] Reagan LP, Flanagan-Cato LM, Yee DK, Ma LY, Sakai RR, Fluharty SJ (1994). Immunohistochemical mapping of angiotensin type 2 (AT_2_) receptors in rat brain. *Brain Research*.

[153] Huang J, Hara Y, Anrather J, Speth RC, Iadecola C, Pickel VM (2003). Angiotensin II subtype 1A (AT_1a_) receptors in the rat sensory vagal complex: subcellular localization and association with endogenous angiotensin. *Neuroscience*.

[154] Wang G, Anrather J, Huang J, Speth RC, Pickel VM, Iadecola C (2004). NADPH oxidase contributes to angiotensin II signaling in the nucleus tractus solitarius. *Journal of Neuroscience*.

[155] Glass MJ, Huang J, Speth RC, Iadecola C, Pickel VM (2005). Angiotensin II AT-1A receptor immunolabeling in rat medial nucleus tractus solitarius neurons: subcellular targeting and relationships with catecholamines. *Neuroscience*.

[156] Wang G, Milner TA, Speth RC (2008). Sex differences in angiotensin signaling in bulbospinal neurons in the rat rostral ventrolateral medulla. *American Journal of Physiology*.

[157] Pierce JP, Kievits J, Graustein B, Speth RC, Iadecola C, Milner TA (2009). Sex differences in the subcellular distribution of angiotensin type 1 receptors and NADPH oxidase subunits in the dendrites of C1 neurons in the rat rostral ventrolateral medulla. *Neuroscience*.

[158] Coleman CG, Anrather J, Iadecola C, Pickel VM (2009). Angiotensin II type 2 receptors have a major somatodendritic distribution in vasopressin-containing neurons in the mouse hypothalamic paraventricular nucleus. *Neuroscience*.

[159] Wang G, Coleman CG, Glass MJ (2012). Angiotensin II type 2 receptor-coupled nitric oxide production modulates free radical availability and voltage-gated Ca2+ currents in NTS neurons. *American Journal of Physiology*.

[160] Gao L, Zucker IH (2011). AT_2_ receptor signaling and sympathetic regulation. *Current Opinion in Pharmacology*.

[161] Zucker IH, Schultz HD, Patel KP, Wang W, Gao L (2009). Regulation of central angiotensin type 1 receptors and sympathetic outflow in heart failure. *American Journal of Physiology*.

[162] Gao L, Wang W, Li YL (2005). Sympathoexcitation by central ANG II: roles for AT_1_ receptor upregulation and NAD(P)H oxidase in RVLM. *American Journal of Physiology*.

[163] Gao L, Wang W, Wang W, Li H, Sumners C, Zucker IH (2008). Effects of angiotensin type 2 receptor overexpression in the rostral ventrolateral medulla on blood pressure and urine excretion in normal rats. *Hypertension*.

[164] Merrill DC, Thompson MW, Carney CL (1996). Chronic hypertension and altered baroreflex responses in transgenic mice containing the human renin and human angiotensinogen genes. *Journal of Clinical Investigation*.

[165] Xia H, Feng Y, Obr TD, Hickman PJ, Lazartigues E (2009). Angiotensin II type 1 receptor-mediated reduction of angiotensin-converting enzyme 2 activity in the brain impairs baroreflex function in hypertensive mice. *Hypertension*.

[166] Rodriguez-Perez AI, Valenzuela R, Villar-Cheda B, Guerra MJ, Lanciego JL, Labandeira-Garcia JL (2010). Estrogen and angiotensin interaction in the substantia nigra. Relevance to postmenopausal Parkinson’s disease. *Experimental Neurology*.

[167] Rodriguez-Pallares J, Rey P, Parga JA, Muñoz A, Guerra MJ, Labandeira-Garcia JL (2008). Brain angiotensin enhances dopaminergic cell death via microglial activation and NADPH-derived ROS. *Neurobiology of Disease*.

[168] Joglar B, Rodriguez-Pallares J, Rodriguez-Perez AI, Rey P, Guerra MJ, Labandeira-Garcia JL (2009). The inflammatory response in the MPTP model of Parkinson’s disease is mediated by brain angiotensin: relevance to progression of the disease. *Journal of Neurochemistry*.

[169] Garrido-Gil P, Valenzuela R, Villar-Cheda B, Lanciego JL, Labandeira-Garcia JL (2012). Expression of angiotensinogen and receptors for angiotensin and prorenin in the monkey and human substantia nigra: an intracellular renin-angiotensin system in the nigra. *Brain Structure and Function*.

[170] Speth RC, Barry WT, Smith MS, Grove KL (1999). A comparison of brain angiotensin II receptors during lactation and diestrus of the estrous cycle in the rat. *American Journal of Physiology*.

[171] Zelezna B, Richards EM, Tang W, Lu D, Sumners C, Raizada MK (1992). Characterization of a polyclonal anti-peptide antibody to the angiotensin II type-1 (AT_1_) receptor. *Biochemical and Biophysical Research Communications*.

[172] Guo DF, Chenier I, Tardif V, Orlov SN, Inagami T (2003). Type 1 angiotensin II receptor-associated protein ARAP1 binds and recycles the receptor to the plasma membrane. *Biochemical and Biophysical Research Communications*.

[173] Deslauriers B, Ponce C, Lombard C, Larguier R, Bonnafous JC, Marie J (1999). N-glycosylation requirements for the AT_1a_ angiotensin II receptor delivery to the plasma membrane. *Biochemical Journal*.

[174] AbdAlla S, Lother H, Quitterer U (2000). AT_1_-receptor heterodimers show enhanced G-protein activation and altered receptor sequestration. *Nature*.

[175] Cook JL, Re RN, DeHaro DL, Abadie JM, Peters M, Alam J (2008). The trafficking protein GABARAP binds to and enhances plasma membrane expression and function of the angiotensin II type 1 receptor. *Circulation Research*.

[176] McKinley MJ, Badoer E, Oldfield BJ (1992). Intravenous angiotensin II induces Fos-immunoreactivity in circumventricular organs of the lamina terminalis. *Brain Research*.

[177] Rockman HA, Chien KR, Choi DJU (1998). Expression of a *β*-adrenergic receptor kinase 1 inhibitor prevents the development of myocardial failure in gene-targeted mice. *Proceedings of the National Academy of Sciences of the United States of America*.

[178] Cook JL, Singh A, DeHaro D, Alam J, Re RN (2011). Expression of a naturally occurring angiotensin AT_1_ receptor cleavage fragment elicits caspase-activation and apoptosis. *American Journal of Physiology*.

[179] Benicky J, Hafko R, Sanchez-Lemus E, Aguilera G, Saavedra JM (2012). Six commercially available angiotensin II AT_1_ receptor antibodies are non-specific. *Cellular and Molecular Neurobiology*.

[180] Adams JM, McCarthy JJ, Stocker SD (2008). Excess dietary salt alters angiotensinergic regulation of neurons in the rostral ventrolateral medulla. *Hypertension*.

[181] Herrera M, Sparks MA, Alfonso-Pecchio AR, Coffman LMTM (2013). Lack of specificity of commercial antibodies leads to misidentification of Angiotensin type 1 receptor protein. *Hypertension*.

[182] Michel MC, Wieland T, Tsujimoto G (2009). How reliable are G-protein-coupled receptor antibodies?. *Naunyn-Schmiedeberg’s Archives of Pharmacology*.

[183] Xue B, Beltz TG, Yu Y (2011). Central interactions of aldosterone and angiotensin II in aldosterone- and angiotensin II-induced hypertension. *American Journal of Physiology*.

[184] Ito M, Oliverio MI, Mannon PJ (1995). Regulation of blood pressure by the type 1A angiotensin II receptor gene. *Proceedings of the National Academy of Sciences of the United States of America*.

